# The need for mathematical modelling of spatial drug distribution within the brain

**DOI:** 10.1186/s12987-019-0133-x

**Published:** 2019-05-16

**Authors:** Esmée Vendel, Vivi Rottschäfer, Elizabeth C. M. de Lange

**Affiliations:** 1 Mathematical Institute, Leiden University, Niels Bohrweg 1, 2333CA Leiden, The Netherlands; 2 Leiden Academic Centre for Drug Research, Einsteinweg 55, 2333CC Leiden, The Netherlands

**Keywords:** Mathematical modeling, Drug transport, Brain extracellular fluid, Blood–brain barrier, Pharmacokinetics

## Abstract

The blood brain barrier (BBB) is the main barrier that separates the blood from the brain. Because of the BBB, the drug concentration-time profile in the brain may be substantially different from that in the blood. Within the brain, the drug is subject to distributional and elimination processes: diffusion, bulk flow of the brain extracellular fluid (ECF), extra-intracellular exchange, bulk flow of the cerebrospinal fluid (CSF), binding and metabolism. Drug effects are driven by the concentration of a drug at the site of its target and by drug-target interactions. Therefore, a quantitative understanding is needed of the distribution of a drug within the brain in order to predict its effect. Mathematical models can help in the understanding of drug distribution within the brain. The aim of this review is to provide a comprehensive overview of system-specific and drug-specific properties that affect the local distribution of drugs in the brain and of currently existing mathematical models that describe local drug distribution within the brain. Furthermore, we provide an overview on which processes have been addressed in these models and which have not. Altogether, we conclude that there is a need for a more comprehensive and integrated model that fills the current gaps in predicting the local drug distribution within the brain.

## Introduction

The blood–brain barrier (BBB) separates the blood from the brain. The BBB is formed by the brain capillary endothelial cells that constitute the walls of the brain capillaries. Multiprotein complexes called tight junctions are located between adjacent brain capillary endothelial cells and seal the intercellular space, thereby limiting intercellular diffusion. In addition, transport across the BBB is affected by transporters and helper molecules located at the brain capillary endothelial cells that move compounds from the blood to the brain or from the brain to the blood. Consequently, the drug concentration-time profile in the brain may be substantially different from that in the blood [1]. Once in the brain, the drug is subject to distribution and elimination processes: diffusion, bulk flow of the brain extracellular fluid (ECF), extra-intracellular exchange, bulk flow of the cerebrospinal fluid (CSF) and metabolism. Furthermore, the drug may bind to specific binding sites (targets) and non-specific binding sites (brain tissue components). Consequently, the drug concentration-time profile in the brain may be substantially different from that in the blood [1], while also local differences in drug concentration-time profiles within the brain may arise. The local concentration-time profiles within the brain are highly important, since drug effects within the central nervous system (CNS) are driven by the concentration-time profile of a drug at the site of its target: the drug needs to be distributed to its target in sufficient concentrations and duration in order to optimally interact with its target and elicit the desired effect. To predict a drug’s effect, therefore, a quantitative understanding is needed on brain target site distribution. However, as the human brain is inaccessible for sampling, measuring drug concentration-time profiles is highly restricted. Mathematical models are a helpful tool to describe and understand the impact of processes that govern drug distribution within the brain. Moreover, while direct measurement of spatial drug distribution within the brain is restricted, mathematical models allow the *prediction* of the spatial distribution of a drug within the brain. To adequately predict the drug distribution of a drug into and within the brain, a mathematical model should include all of the above mentioned factors that govern the concentration-time profiles of a drug within the brain. However, currently existing models focus on just one or a few of these processes. The aim of this review is to provide an overview of the current state of the art in modelling drug distribution into and within the brain and highlight the need for novel methods that provide a more complete description of the local drug distribution within the brain. We first summarise the factors affecting the drug distribution within the brain (in “Factors affecting drug distribution within the brain”). Then, we give an overview of currently available models on the distribution of compounds into and within the brain and of models that integrate two or more of these aspects (in “Existing models on the local distribution of drugs in the brain”). Finally, in “The need for a refined mathematical model on spatial drug distribution within the brain”, we discuss how we can improve or combine current models to develop a comprehensive model for improved prediction of drug distribution into and within the brain.

## Factors affecting drug distribution within the brain

The distribution of a drug within the brain determines the local concentration of drug that is available to bind to its target and thereby induce an effect. Both the structural properties of the brain and those of the drug affect the distribution of the drug within the brain. In this section, we first discuss the brain-specific and drug-specific properties. Then, we describe the processes that affect local drug distribution within the brain. These processes depend on both the brain-specific and drug-specific properties. Finally, we discuss how spatial variations in drug distribution processes may lead to spatial differences in drug concentration-time profiles within the brain.

### Brain-specific properties

**Fig. 1:**
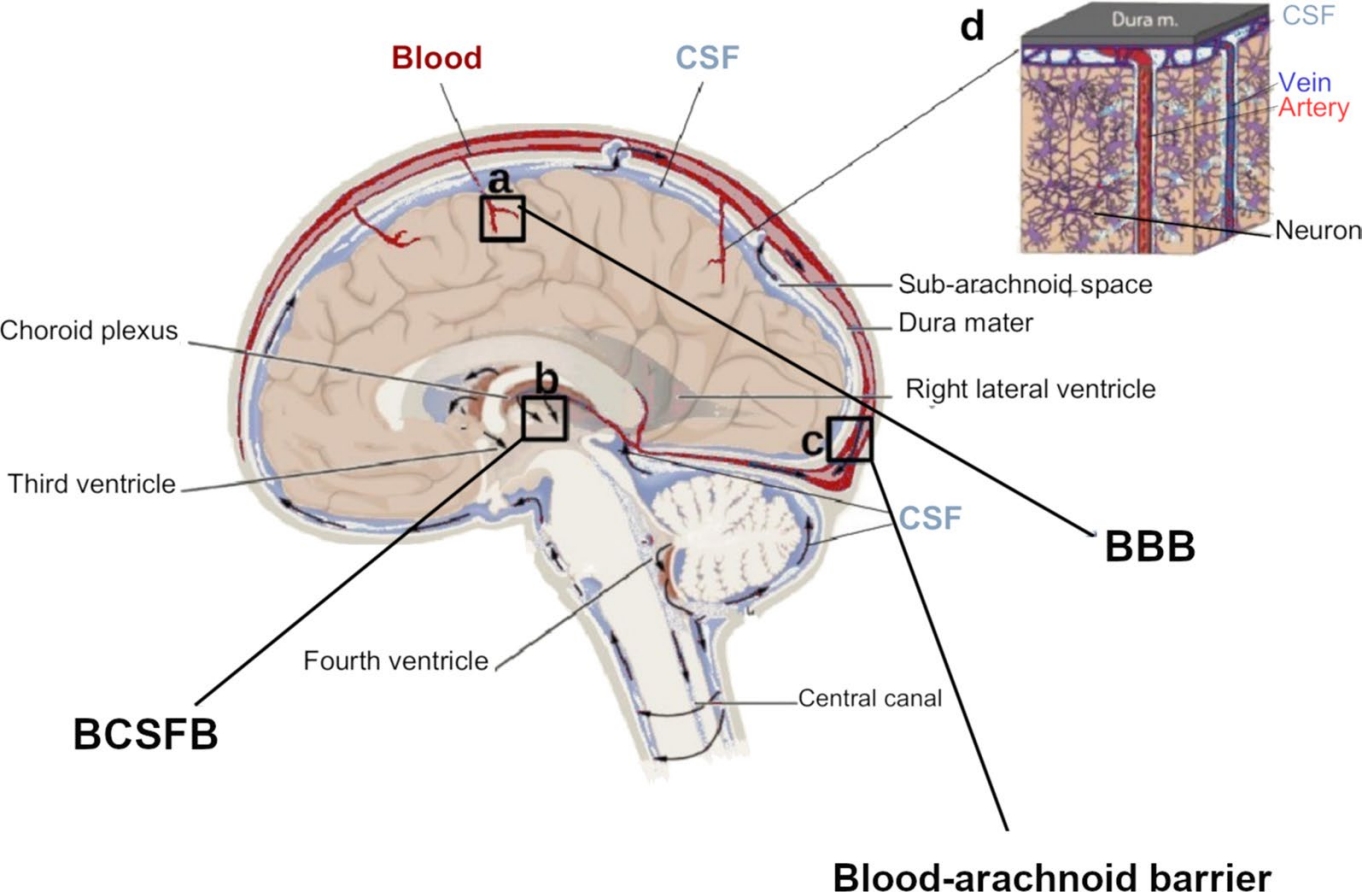
Structure of the human brain: blood, brain tissue, CSF and the brain barriers. Blood vessels (red) infiltrate the brain tissue (grey) and branch out into smaller brain capillaries (inset). At the level of the brain capillaries compounds exchange between the blood and the brain tissue through the BBB. The brain tissue (brain parenchyma) contains the brain cells and the brain ECF. The CSF (blue) is located in the sub-arachnoid space (located between the dura mater, a layer of connective tissue surrounding the brain tissue, and the brain tissue), the brain ventricles and the spine. The blood is separated from the CSF by the BCSFB and the blood–arachnoid barrier. The brain barriers are indicated by black squares **a**–**c**. **a** The BBB is the barrier between the blood in the brain capillaries and the brain tissue. **b** The BCSFB is the barrier between the blood in the capillaries and the CSF in the brain ventricles. **c** The blood–arachnoid barrier is the barrier between the blood in the blood vessels of the dura mater and the CSF in the sub-arachnoid space. **a**–**c** are adapted from [242] and licensed under CC BY 4.0. **d** is adapted with permission from [243]

The brain-specific properties are the structural properties of the brain. The structural properties most important for drug distribution within the brain are highlighted in Fig. 1. Blood is supplied to the brain by arteries feeding the anterior (front) or posterior (back) part of the brain. The arteries branch out into smaller brain capillaries. At the level of the brain capillaries, compounds are exchanged between the blood and the brain tissue. The blood in the brain capillaries is separated from the brain tissue by the BBB (Fig. 1a). The brain capillaries reunite to form veins, from which the blood is carried away from the brain back into the heart. The brain tissue (brain parenchyma) consists of the brain ECF and the brain cells. The brain ECF surrounds the cells and circulates within the brain tissue. The CSF circulates between the sub-arachnoid space (located between the dura mater, a layer of connective tissue surrounding the brain tissue, and the brain tissue, see Fig. 1), the brain ventricles, and the spine (Fig. 1). The blood is separated from the CSF by the blood–CSF barrier (BCSFB) and the blood–arachnoid barrier. The BCSFB is located between the blood in the brain capillaries and the CSF in ventricles of the brain (Fig. 1b). The blood–arachnoid barrier is positioned between the blood in the dura mater and the CSF in the sub-arachnoid space (Fig. 1c). The entire brain contains many potential binding sites for endogenous compounds (that originate within the body) and exogenous compounds (that originate outside the body). In addition, metabolic enzymes residing in the brain may chemically convert substances into new molecules. In the subsequent sections, we highlight the properties of the brain vascular network, the brain barriers, the brain tissue (including the brain ECF and the brain cells), the CSF, the fluid movement within the brain, binding and metabolism.

#### The brain vascular network

**Fig. 2:**
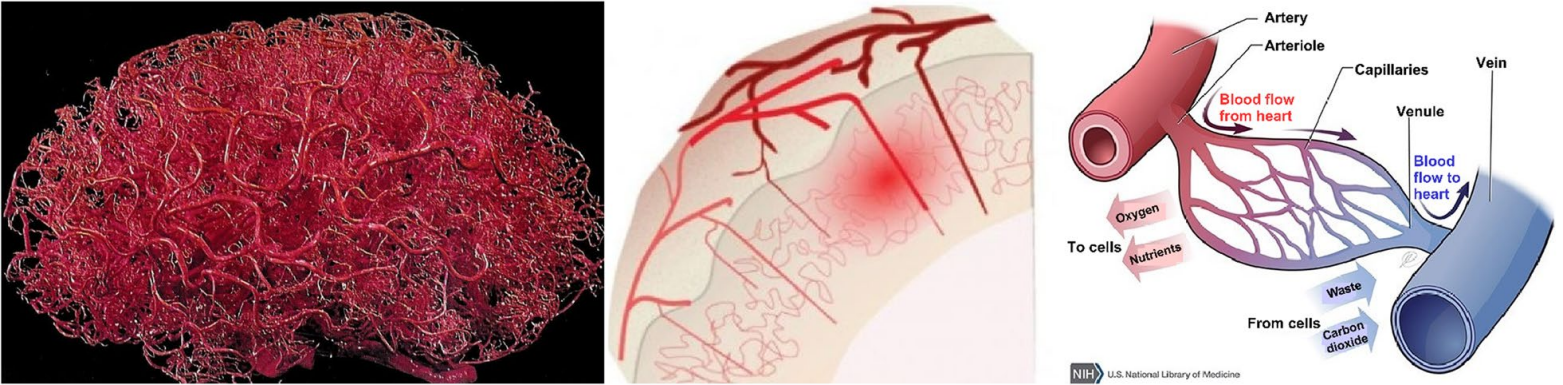
The brain vascular network. Left: the network of brain vasculature (public domain image) [245]. Middle: vascular organization of the cerebral cortex [97]. Arterioles (pink) penetrate the brain cortex and branch out into dense capillary beds that feed an active region of the brain (highlighted in red). Venules (dark red) take away the blood from the brain capillary beds. The image by by [97] is licensed under CC BY 3.0. Right: capillaries. A capillary bed consists of a small network of capillaries. The brain capillaries are fed with oxygen and nutrients by the blood flow from the general blood circulation through the arteries and arterioles. Waste products are carried away from the brain capillaries by blood flow back into the heart through the venules and veins. Adapted with permission from [244]

An extensive network of vasculature supplies the brain with oxygen and nutrients (Fig. 2, left). The brain surface is perfused with large arteries and veins that carry oxygen and nutrients to the brain (Fig. 2, middle). The larger brain arteries branch out into smaller arterioles that penetrate the brain cortex and merge into the brain microcirculation, consisting of the brain capillary beds (Fig. 2, right). The brain capillaries that make up the capillary beds surround the brain tissue. Waste products are carried away from the capillary beds by the venules. The venules merge into the veins, which lead the blood and the waste products it contains back to the heart. The brain capillaries have a large surface area: they are the main site for the exchange of oxygen and nutrients with the brain tissue [2]. The brain capillary network is very dense and it is estimated that each neuron is perfused with its own capillary [3]. The average distance between the capillaries in the rat brain is only about 50 $\upmu \text {m}$ [4–7]. The brain capillaries are separated from the brain by the brain barriers, which will be discussed in the next section.

#### The barriers of the brain

Three barriers are known that separate the blood in the brain capillaries from the brain:

1. The BBB, which separates the blood in the brain capillaries from the brain tissue, including the brain ECF and the brain cells.
2. The BCSFB, which separates the blood in the brain capillaries from the CSF in the brain ventricles.
3. The blood–arachnoid barrier, which separates the blood in the blood vessels of the dura mater from the CSF in the sub-arachnoid space (see Fig. 1).

**Fig. 3:**
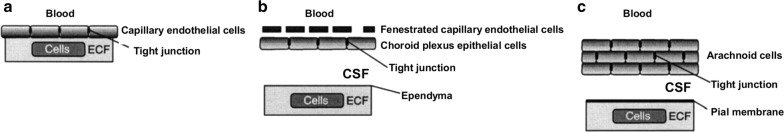
Barriers of the brain. **a** The BBB. The BBB separates the blood from the brain tissue, including the brain ECF and the cells. The barrier exists at the level of the brain capillary endothelial cells, which are connected by tight junctions. **b** The BCSFB. The BCSFB separates the blood from the CSF in the brain ventricles. The barrier function exists at the level of the choroid plexus epithelial cells, that are connected by tight junctions. Unlike at the BBB, the capillaries between the blood and the CSF are fenestrated (contain pores) and are not connected by tight junctions. A layer of cells of the ependyma separates the CSF from the brain ECF. **c** The arachnoid barrier. The arachnoid barrier separates the blood in the blood vessels of the dura mater from the CSF in the sub-arachnoid space. The barrier function is exerted by the arachnoid cells, that are connected by tight junctions. A layer of cells of the pia mater (pial cells) separates the CSF from the brain ECF. Adapted with permission from [2]

The main characteristics of each barrier are summarised in Fig. 3 and described below. Drug transport across these brain barriers is described later in “Processes affecting drug distribution within the brain”.

##### The BBB

The BBB protects the brain against the influx of toxic or harmful substances [8]. Moreover, it helps maintaining brain homeostasis by regulating the transport of ions, molecules and leukocytes into and out of the brain [9]. The BBB separates the blood from the brain and consists of the brain endothelial cells, that constitute the walls of the brain capillaries. Depending on the drug, transport across the BBB might be more or less difficult. Typically, the brain endothelial cells form a firmly closed layer of cells [10] (Fig. 3a). Tight junctions, multiprotein complexes located in the narrow space between the brain endothelial cells, and a lack of fenestrations (small pores) between adjacent brain capillary endothelial cells make it hard for compounds to pass through the intercellular space [11]. Around the brain endothelial cells, astrocytes (supportive cells, see “The brain tissue and the CSF”) connect with neurons and pericytes, the latter regulating the BBB functionality [8]. Together, they form the so-called neurovascular unit, which is the actual barrier of the brain.

##### The BCSFB

The BCSFB separates the blood in the brain capillaries from the CSF. It regulates the exchange of compound in order to maintain a stable environment for normal brain function. The barrier consists of the epithelial cells of the choroid plexus located in the brain ventricles (Fig. 1). These cells are strongly connected by tight junctions (Fig. 3b). In contrast, the brain capillaries of the BCSFB are, unlike those of the BBB, fenestrated (contain pores) and highly permeable.

##### The blood–arachnoid barrier

The blood–arachnoid barrier separates the (fenestrated) brain capillaries in the dura mater from the CSF in the sub-arachnoid space (see Fig. 1) [12–14]. The barrier is formed by a layer of arachnoid cells (epithelial cells located between the dura mater and the sub-arachnoid space), that are connected by tight junctions (Fig. 3c).

#### The brain tissue and the CSF

The brain tissue consists of the brain ECF and the cells containing intracellular fluid (ICF). It is perfused with the brain vasculature (see also “The brain vascular network”) and surrounded by the CSF. The properties of the brain ECF, brain cells and CSF will be discussed below.

##### The brain ECF

The brain ECF surrounds the brain cells and occupies about $20\%$ of the brain tissue. It is also referred to as the brain interstitial fluid to avoid confusion with the blood plasma, which is in fact also an ECF. The brain ECF is crucial in the transport of both endogenous and exogenous compounds [15, 16]. It is produced by filtration of blood plasma through the brain capillary walls that constitute the BBB. As proteins cannot pass the BBB, the composition of the brain ECF is similar to that of the blood plasma but includes a minimal amount of proteins.

##### The brain cells

The brain cells can be classified into neurons, supportive cells (glial cells) and pericytes. Neurons are excitable brain cells that transmit information by electrical and chemical impulses. They have a typical morphology, consisting of one long axon and one or multiple shorter dendrites attached to the cell body. Multiple axons can be packed together in so called nervous tracts. The glial cells support and protect the neurons and include astrocytes, oligodendrocytes and microglia [17]. Of these, astrocytes have an important function in regulating local blood flow to match the transport of oxygen and nutrients to neuronal activity [17–21]. The astrocytes are in contact with both brain endothelial cells and neurons. Finally, pericytes surround the brain endothelial cells and help regulate the permeability of the BBB and the brain capillary blood flow by contraction movements [17]. Together, the brain cells make up almost $80\%$ of the volume of the brain tissue [17]. Cellular composition differs between the white matter in the deep parts of the brain and the grey matter in the more superficial parts of the brain. The white matter consists mostly of nervous tracts, that contain long, myelinated axons branching out from neurons. Myelination refers to the insulation of axons by myelin to speed up the transmission of information along the nervous tracts. The grey matter consists of neurons (the cell body, dendrites and unmyelinated axons), glial cells, and brain capillaries.

##### The CSF

The CSF resides in the four brain ventricles and in the sub-arachnoid space. It provides a mechanical protection of the brain (against shocks and injuries), helps in the discard of waste and compensates blood volume changes in the brain during the cardiac cycle [22]. The CSF is mostly produced by the epithelial cells of the choroid plexus in the ventricles of the brain [23] (Fig. 1). Recently, it has been hypothesised that the CSF is produced within the entire brain CSF circulation, as a result of the filtration of fluid across the brain capillary walls into the brain ECF [24]. The CSF is a clear fluid with a low protein concentration with similar composition as the brain ECF.

#### Fluid movements within the brain

Regular recycling and clearance of the brain ECF is needed for maintaining homeostasis within the brain tissue [25]. The ependymal cell layer between the brain ECF and the CSF in the brain ventricles (see Fig. 3b) and the pial cell layer between the brain ECF and the CSF in the sub-arachnoid space (see Fig. 3c) are both relatively permeable. Hence, fluid freely circulates between the brain ECF and the CSF [26, 27]. The movement of both the brain ECF and the CSF will be discussed below.

##### Brain ECF movement

The brain ECF is produced by the secretion of fluid from the brain capillary endothelial wall. This arises from the passive movement of water across the BBB in response to ionic gradients [25]. Within the brain, the brain ECF moves through the extracellular space by the brain ECF bulk flow. The brain ECF bulk flow is driven by hydrostatic pressure [27, 28] or pulsatile movements of the brain arteries [29]. The brain ECF bulk flow is directed towards the CSF in the ventricles and in the sub-arachnoid space. There, the CSF acts as a sink because of its turnover (see “CSF movement”) [30]. Alternatively, the brain ECF may drain directly across the capillary and arterial walls into the lymphatic system [30]. The importance of the brain ECF bulk flow relative to diffusion has been under debate [27, 31, 32]. A recently proposed “glymphatic mechanism“ describes the convective fluid transport from the para-arterial to para-venous space through the brain ECF that is regulated by the glia cells [23, 29, 33, 34]. This “glymphatic mechanism” derives its name from its dependence on glial cells and its resemblance to the removal of waste products by lymph systems outside of the brain [35, 36]. It involves the exchange of fluid between the brain ECF and the CSF, in which the CSF enters the brain ECF from the arteries or arterioles, while the brain ECF exits along the veins or venules [33, 36]. This fluid exchange is suggested to depend on so-called aquaporin-4 channels that are located at the astrocyte endfeet and facilitate the transport of water across barriers [33, 35–37]. The “glymphatic mechanism” lacks a mechanistic basis and therefore, mathematical modelling comes into use. Recent modelling studies taking the “glymphatic mechanism” of brain ECF bulk flow into account demonstrate that transport within the brain ECF is dominated by diffusion [38, 39]. Despite the controversy around the importance of bulk flow within the brain ECF relative to diffusion, there is evidence that the brain ECF bulk flow affects brain diseases, including epilepsy [25].

##### CSF movement

The CSF is produced by the epithelial cells of the choroid plexus that constitute the BCSFB (Figs. 1b and 3b). The CSF is generally assumed to circulate between the brain ventricles and sub-arachnoid space before reabsorption into the blood of the peripheral blood stream at the level of the arachnoid membrane (Fig. 1c). The CSF can also be absorbed into the lymphatic system [40]. Part of the CSF can be absorbed into the brain tissue via the Virchow–Robin space (fluid-filled canals around the blood vessels that penetrate the brain tissue) or the para-arterial space [25, 31, 41–43]. There is evidence that the Virchow-Robin space functions as a drainage pathway for the clearance of waste molecules from the brain and is also a site of interaction between the brain and the (systemic) immune system [23]. A new view starts to emerge that considers the CSF to be produced within the entire CSF system and describes the CSF circulation as much more complicated [23–25]. There, the CSF circulation includes directed CSF bulk flow, pulsatile back-and-forth movements of fluid between the brain ECF and CSF and the continuous bidirectional exchange of fluid across the BBB and the cell layers between the brain ECF and CSF (see Fig. 3) [23].

#### Metabolic enzymes

Metabolic enzymes chemically alter substances into new molecules, the metabolites. Important metabolic enzymes include the cytochrome P450 proteins and conjugating enzymes [44]. The liver is the main site for (drug) metabolism and contains high concentrations of cytochrome P450 proteins. In the brain, cytochrome P450 proteins are also present. Particularly in and around the cerebral blood vessels and the brain barriers, cytochrome P450 and conjugating enzymes have been detected [45]. Even though in the brain the cytochrome P450 proteins exist at much lower levels than in the liver, they may substantially affect local metabolism depending on their location [46].

### Drug-specific properties

**Fig. 4:**
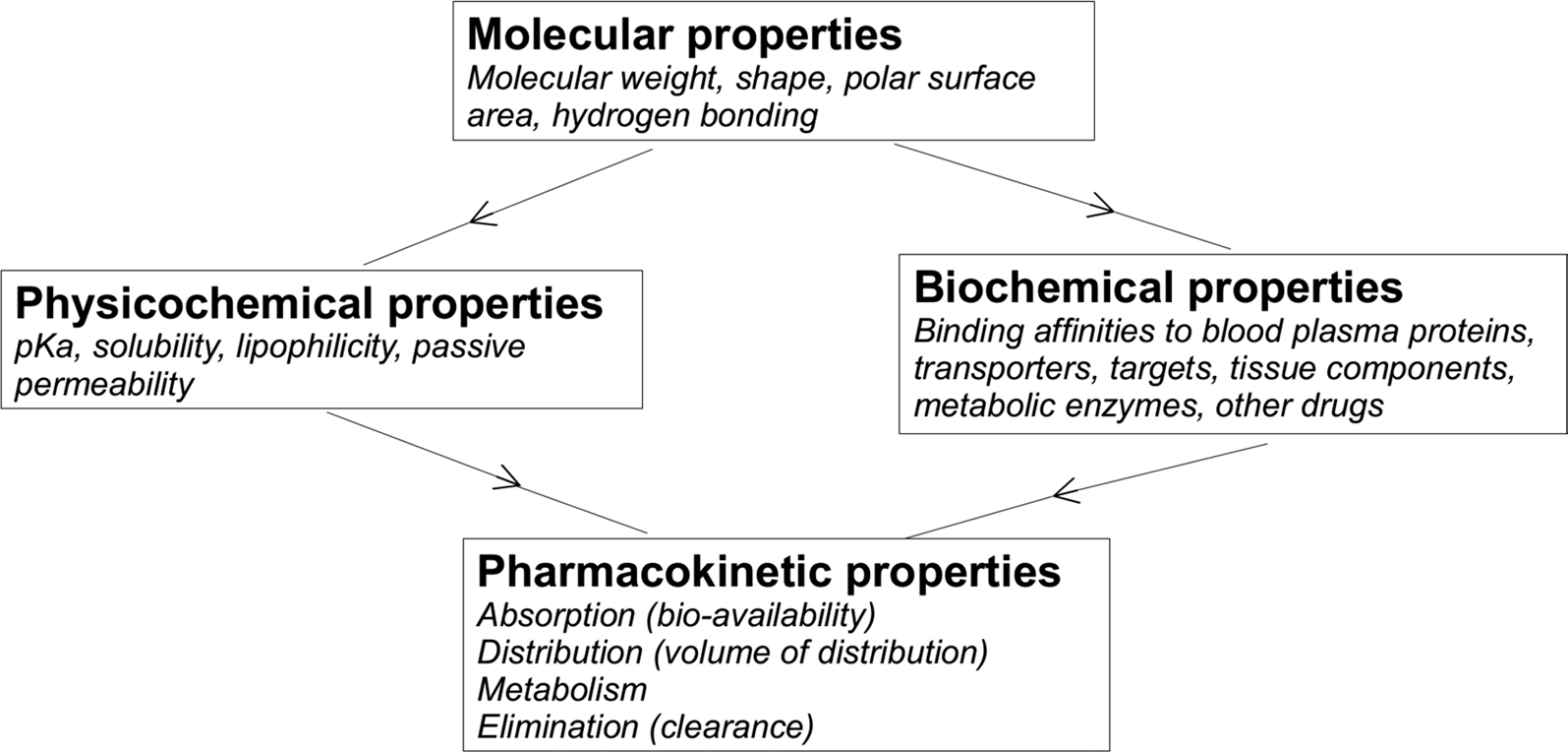
The properties of a drug affecting its distribution within the brain. The molecular properties are the properties inherent to the drug and affect both its physicochemical and biochemical properties. The physicochemical properties describe the interaction of a drug with its physical environment, while the biochemical properties describe the binding affinities of a drug to other molecules. The pharmacokinetic properties depend on both the physicochemical and biochemical properties

The properties of a drug affect its distribution within the brain. These properties can be classified into molecular properties inherent to the drug and other properties that emerge from the interaction of the drug with its environment. These include the physicochemical properties and binding affinities, that in turn affect the pharmacokinetic properties (Fig. 4). All are discussed below.

#### Molecular properties

The molecular properties of the drug are the most basic properties inherent to the drug. The most important structural properties are:

- The *molecular weight*. This is the mass of one molecule of the drug. The molecular weight correlates with absorption, diffusion, transport across the BBB, but also with active transport back into the blood [47]. A low molecular weight is usually related to a better distribution into and within the brain. Most drugs that diffuse through the BBB have a molecular weight below 500 [48].
- The *shape*. This is the outline of the space occupied by the drug and can highly influence the interactions of a drug with its environment (see [49] for a review).
- The *polar surface area*. This is the surface area occupied by all polar (generally nitrogen and oxygen) atoms of the drug. This is important as the polar atoms of a drug are involved in the transport between aqueous (polar) and membrane (non-polar) regions. To diffuse through the BBB, the polar surface area usually needs to be less than 90 $\mathring{\mathrm{A}}^2$ [50].
- The number of *hydrogen bond donors and acceptors*. Hydrogen bonds are weak bonds resulting from electrostatic interactions between a hydrogen atom bound to a more electronegative atom (the donor) and another electronegative atom (the acceptor). The number of hydrogen bond donors and acceptors in a drug molecule determines the likeliness of a drug to take part in hydrogen bonding with molecules in its environment (see “Drug-specific properties that depend on the environment” section).

#### Drug-specific properties that depend on the environment

The molecular properties of a drug affect how a drug interacts with its environment. (Fig. 4). Properties of drug interaction with its environment can be classified into:

- The *physicochemical properties*. These determine the interaction of a drug with the environment it resides in, including the fluid and tissue components. Important examples of physicochemical properties are:
  - The *pKa*. This is the pH (of the environment) at which the drug exists for $50\%$ in its charged state and for $50\%$ in its uncharged state. As the pH of the body is limited to a narrow range, the pKa of the drug greatly affects its charge. In turn, the charge of a drug affects many factors, including the drug solubility, lipophilicity, binding affinities and pharmacokinetic properties (see “Pharmacokinetic properties” below). While charged drugs generally have a higher solubility, uncharged drugs are more lipophilic and therefore cross cell membranes more easily [47].
  - The *solubility*. This is the ability of a drug to dissolve in the environment it resides in to give a homogeneous system. This is crucial for drug absorption: in order to be absorbed, drug needs to be fully dissolved at the site of absorption.
  - The *lipophilicity*. This describes how easily a drug dissolves in non-polar (i.e. ‘fatty’) solvents compared to in polar solvents, like water. It can be estimated using log *P*_oct/wat_, which is the log of the ratio of the drug dissolved in octanol and drug dissolved in water, at a pH for which all drug molecules are non-charged. A drug’s lipophilicity is highly important for drug transport across the (lipophilic) cell membranes.
- The *biochemical properties*. These determine the interaction of a drug with proteins and other molecules and affect the concentration of free drug. They include drug binding affinities to blood plasma proteins, drug targets, transporters, tissue components and metabolic enzymes. Therewith, binding greatly impacts the pharmacokinetic properties of the drug (see “Pharmacokinetic properties” below). The interaction of a drug with binding sites not only depends on strong, covalent binding (when a pair of electrons is shared between two atoms), but can also be greatly affected by weaker hydrogen bonds.

#### Pharmacokinetic properties

The pharmacokinetic properties depend on both the physicochemical and biochemical properties of the drug. They quantify the disposition of a drug, which refers to its absorption, distribution, metabolism and elimination (also known by the acronym ADME):

- Absorption generally refers to drug absorption into the systemic circulation (the blood). The *bio-availability* is a common measure for the fraction of drug that is absorbed into the blood.
- Distribution includes drug transport across barriers, drug transport within fluids (e.g. by diffusion), intra-extracellular exchange and drug binding. The *volume of distribution* defines the distribution of drug between the blood plasma and the rest of the body. Drugs that highly distribute into tissues, i.e. by exchange with cells or binding to tissue components, or drugs that have a low extent of plasma protein binding, generally have a high volume of distribution.
- Metabolism of a drug depends on the concentration of metabolic enzymes, the maximal velocity of the metabolic reaction mediated by the enzymes and the interaction of a drug with the metabolic enzymes (see “Drug-specific properties that depend on the environment” above).
- Elimination of a drug generally refers to the processes by which a drug is cleared from the body. Common pharmacokinetic properties related to drug elimination are the drug elimination *clearance* and drug *half-life*.

Definitions of the discussed pharmacokinetic properties (*italic*) are given below.

**Table:** 

Bioavailability = The fraction of drug that enters the systemic circulation unchanged or the rate and extent at which drug enters the systemic circulation	
Half-life = The time needed for the concentration of drug to be reduced by a half	
Elimination clearance = The rate at which active drug is removed from the brain	
Volume of distribution = The apparent volume that is required to keep the drug at the same concentration as is observed in the blood plasma	

### Processes affecting drug distribution within the brain

**Fig. 5:**
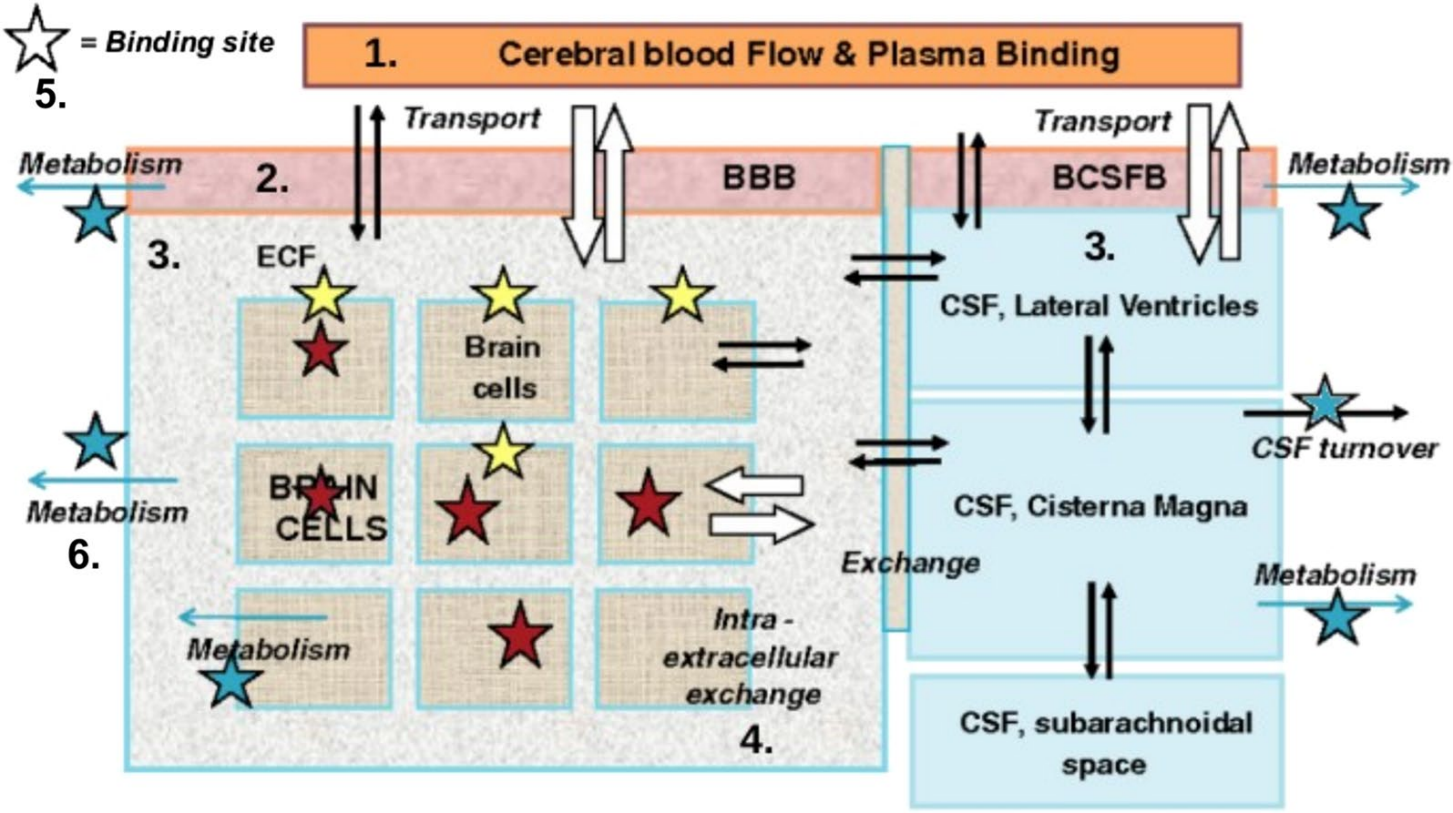
Schematic presentation of the major compartments of the mammalian brain and routes for drug exchange [45]. 1: drug transport through the brain vascular system. 2: drug transport across the brain barriers, including the BBB and the BCSFB. 3: drug transport within the brain fluids (brain ECF and CSF). 4: drug intra-extracellular exchange. 5: drug binding to binding sites that may be intracellular (brown stars), extracellular (yellow stars) or metabolic enzymes (blue stars). Drug binding sites may be present at different sites within the brain. 6: drug metabolism by metabolic enzymes (blue stars). Black arrows: passive transport. White arrows: active transport. Blue arrows: metabolic reactions. The image by [45] is licensed under CC BY 2.0 and modified for the purpose of this review

A drug needs to be distributed to its target area in sufficient concentrations in order to interact with its target and exert the desired effect. Drug distribution is affected by both the brain-specific and drug-specific properties discussed in the previous subsections. Within the brain, the *unbound* drug is exchanged between several components, including the blood plasma, the brain ECF, the CSF and the brain cells. The unbound drug therefore drives the distribution of drug into and within the brain [45, 51]. In order to provide a qualitative understanding of the processes that are related to drug distribution we summarise the relevant processes in this subsection. For this purpose, we give a schematic representation in Fig. 5. We make the following classification of processes related to drug distribution within the brain:

1. Drug transport through the brain vascular system.
2. Drug transport across the brain barriers.
3. Drug transport within the brain fluids.
4. Drug extra-/intracellular exchange.
5. Drug binding.
6. Drug metabolism.

The corresponding numbers can be found in Fig. 5. Below, we provide a description of each of these processes.

#### Drug transport through the brain vascular system

Drugs within the cerebral circulation are first transported by the cerebral blood flow in the larger blood vessels and finally presented to the brain by the brain capillary blood flow in the microcirculation (i.e. the brain capillaries). Hence, blood flow velocity is important for drug delivery to the brain. In the large arteries and veins, the blood flow rate is about 750 mL min^−1^. However, within the brain capillaries, where drug is exchanged with the brain tissue, the capillary blood flow rate is only 6–12 nL min^−1^ [17, 52]. Within the blood, drug may bind to red blood cells or, particularly, blood plasma proteins, such as albumin and $\alpha$1-acid glycoprotein. The percentage of drug that binds to blood plasma proteins varies strongly among drugs and as much as $99.9\%$ of the drug may be protein-bound [53]. This greatly reduces the concentration of (unbound) drug that can cross the brain barriers to get into the brain.

#### Drug transport across the brain barriers

**Fig. 6:**
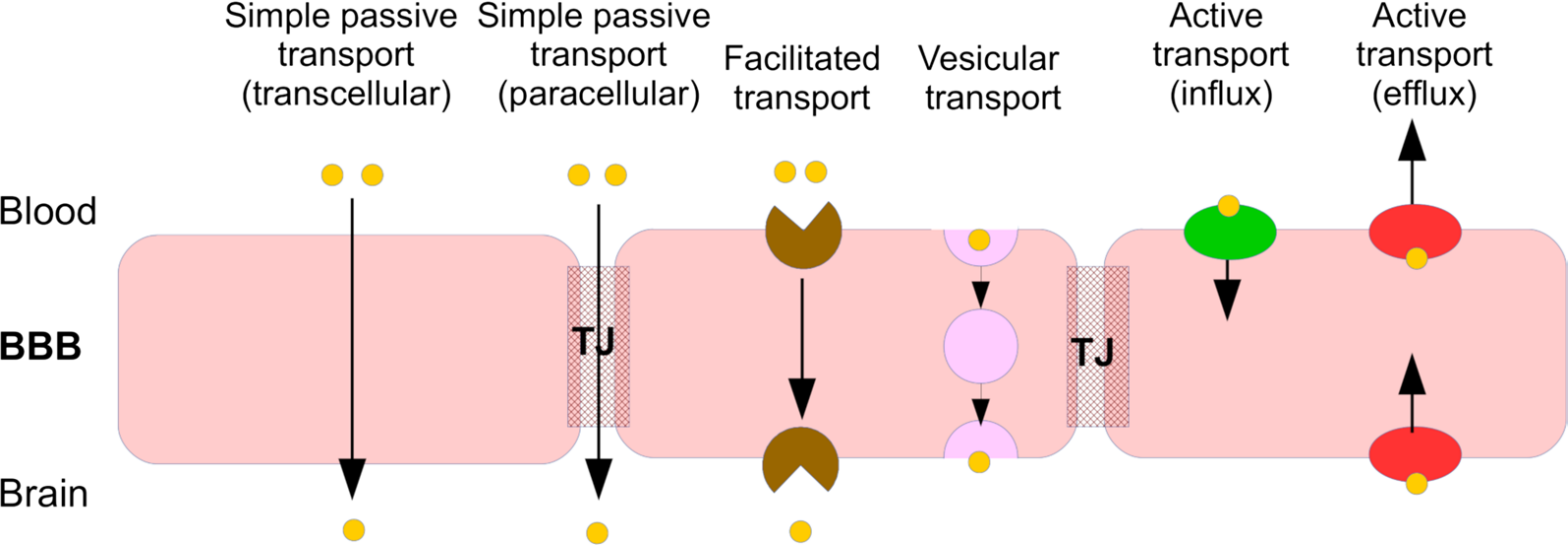
Modes of drug transport across the brain barriers. In the figure, the BBB is shown, but the modes of drug transport also apply to the other barriers. In simple passive transport, drugs cross the BBB (or the other brain barriers) passively through the cells (transcellular) or between the cells (paracellular) by diffusion. In facilitated transport, drug diffusion across the BBB is aided by helper molecules. In vesicular transport, drugs move across the BBB through vesicles that are formed within the barrier. In active transport, drugs are actively transported into the brain by specific influx transporters or out of the brain by efflux transporters. *TJ* tight junction

**Fig. 7:**
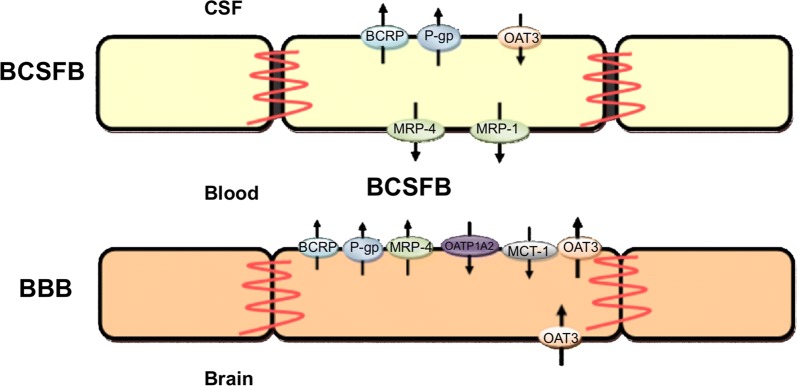
The localization of transporters in the BBB and BCSFB [63]. The BCSFB (top) and BBB (bottom) are shown. Active transporters are located at both sides of the BCSFB, but mostly at the apical (blood-facing) membrane of the BBB. This image by [63] is licensed under CC BY 3.0

Drugs within the blood in the brain capillaries need to cross the brain barriers in order to enter the brain tissue. The movement of drugs from the blood plasma across the brain barriers into the brain tissue involves the crossing of two separated membranes in series. Drug movement across the brain barriers can be classified into several modes of transport as summarised in Fig. 6 and described below:

- *Simple passive transport*, in which drugs diffuse across the barrier by following a concentration gradient between the blood in the brain capillaries and the fluids in the brain. The rate of diffusion is proportional to the drug concentration difference between both sides of the barrier. The ease of drug diffusion across the barrier is determined by the permeability of the barrier to the drug to cross. This permeability depends on both the intrinsic permeability of the barrier (see  “The barriers of the brain” in “Brain-specific properties”) and the molecular characteristics (such as size, shape an charge) of the drug (see “Drug-specific properties”). A drug may diffuse directly through the cells of the barrier (transcellular diffusion) or through the space between the cells (paracellular diffusion). At the BBB, paracellular diffusion is the main route of transport for hydrophilic molecules, that cannot cross the cells. In the healthy brain, paracellular transport is restricted by the presence of the tight junctions in the intercellular space between the BBB endothelial cells. In in vitro experiments, unstirred water layers may form at both the apical (blood-facing) and abluminal (brain-facing) side of the BBB and affect passive transport and thus influence the results [54]. Their presence results in an increased permeability for hydrophilic drugs and a decreased permeability for lipophilic drugs [55, 56].
- *Facilitated transport*, in which the movement across the barrier down a concentration gradient is aided by transport proteins. The availability of these helper molecules is limited and saturation of helper molecules may occur at sufficiently high drug concentrations.
- *Vesicular transport*, in which molecules move through vesicles that are formed within the barrier. The extent of vesicular transport is much higher on the BCSFB than on the BBB [57, 58]. Three known types of vesicular transport exist: fluid-phase endocytosis, adsorptive endocytosis and receptor-mediated endocytosis. Fluid-phase endocytosis, or pinocytosis, is the energy-dependent uptake of ECF by vesicles, taking along any solutes residing in the fluid. In adsorptive endocytosis, positively charged molecules are non-specifically taken up by negatively vesicles based on electrostatic interactions [59, 60]. In receptor-mediated endocytosis, vesicles form after binding of molecules to specific receptors that are then transported across the barrier [61].
- *Active transport*, in which drugs are actively transported into or out of the brain by drug-specific transporters. Active transporters in the brain are membrane-bound transporters that move endogeneous compounds or exogeneous compounds (like drugs) across the brain barriers. In contrast to facilitated transport, this uses up energy and compounds can be transported against the concentration gradient. The affinity of a drug to an active transporter depends on the molecular characteristics of the drug, such as its polarity and molecular surface. Active transport is directional and can be classified into influx transport and efflux transport. Influx transporters help compounds enter the brain, while efflux transporters move compounds out of the brain. Several active transporters are involved in the movement of drugs across the BBB. These include the organic anion-transporting poly-peptide 1A2 (OATP1A2), organic anion transporter 3 (OAT3), monocarboxylate transporter 1 (MCT-1), *P*-glycoprotein (P-gp), breast-cancer-resistance protein (BCRP) and multidrug-resistance-associated proteins 1-9 (MRP-1-9) [62, 63]. At the BBB, most efflux transporters are located at the apical (blood-facing) membrane of the BBB [64], see Fig. 7. At the BCSFB, BCRP and P-gp are located at the apical (CSF-facing) membrane, while MRP is located at the basolateral (blood-facing) membrane (Fig. 7).

Drug transport into the brain can be affected by metabolic enzymes located at the brain barriers, including the cytochrome P450 haemoproteins and uridine 5′-diphospho (UDP)-glucuronosyltransferases [65]. Metabolic enzymes transform active drugs into inactive substances or facilitate their excretion out of the body. As such, they decrease the concentration of active drugs entering the brain. Alternatively, metabolism at the BBB can be beneficial to the drug in case inactive compounds (“pro-drugs”) are converted into active drugs (see [66] for a review on this topic).

#### Drug transport within the brain fluids

The brain fluids are essential for the distribution of a drug within the brain. Drugs are transported to their targets by diffusion and bulk flow within the brain ECF [26, 27] and the CSF [67].

##### Diffusion within the brain ECF

Diffusion of a drug within the brain ECF is hindered by many obstacles and therefore the brain ECF can be considered as a porous medium [68]. In other words, diffusion within the brain ECF is hindered by the brain cells that determine the geometry and width of the brain ECF [69]. The intercellular space occupied by the brain ECF is only tens of nanometres wide, which is much narrower than the diameter of the surrounding brain capillaries. The effective diffusion of a drug within the brain ECF can be further reduced by dead-space microdomains. Dead-space microdomains are void spaces within the brain ECF in which molecules can be temporarily trapped. So far dead-space microdomains have been found in the diseased, but not in the healthy rat brain [70, 71]. In addition to the mentioned geometrical factors that determine the shape of the brain extracellular space, the brain ECF contains various binding sites that reduce drug transport. The binding of a drug to proteins of the extracellular matrix, negatively charged molecules or other molecules within the brain ECF prevent diffusion of (free) drug. Due to all mentioned factors, the effective diffusion of a drug in the brain ECF is much lower than the free diffusion of the same drug in water.

##### Brain ECF bulk flow

The impact of the brain ECF bulk flow on drug distribution within the brain is disputable (see also “Fluid movements within the brain” in “Brain-specific properties”). Some research groups state that the rate of the brain ECF bulk flow is negligible compared to the rate of diffusion, especially on a short distance [69, 72, 73]. However, there is evidence that the brain ECF bulk flow may be a relevant means of drug distribution within the brain [27, 74, 75]. The brain ECF bulk flow is likely most important for drugs with a high molecular weight, for which diffusion in the brain ECF is hindered [38, 76, 77]. The Péclet number is an useful measure to assess the relative importance of drug transport by the brain ECF bulk flow in comparison to drug transport by diffusion [33, 38]. A Péclet number $\ll$ 1 indicates that diffusion dominates, while a higher number indicates that the brain ECF bulk flow is also important. Within the brain ECF, the Péclet numbers range between ${10}^{-3}$ and ${10}^{0}$ which indicates that diffusion dominates [38] (see also “Fluid movements within the brain”).

##### Drug transport within the CSF

Within the CSF, drug is transported via the CSF bulk flow and diffusion. The CSF bulk flow (see “Fluid movements within the brain”) leads to the rapid removal of drug into the blood. The regular renewal, or turnover, of the CSF may reduce drug concentrations within the CSF [78]. Diffusion mediates the entry of a drug from the CSF into the brain tissue [67]. As the rate of diffusion of drug from the CSF into the brain tissue is much slower than the rate of the CSF bulk flow, the transport of drugs from the CSF into the brain tissue is minimal [67].

#### Drug extra/intracellular exchange

Within the brain tissue, a drug may have a preference for the space inside or outside the cells (intracellular or extracellular space), depending on its properties (see “Drug-specific properties”) [79]. Intra-extracellular exchange is relevant for the distribution and subsequent exposure of a drug to its target site [51]. Drugs distribute between the cells and the extracellular space by simple diffusion, but active transport is also possible [80, 81]. Generally, compounds that easily cross the BBB by passive diffusion (i.e. transcellular diffusion) also cross cellular membranes easily. However, a drug may be actively transported across the BBB but not across the membrane of the cells within the brain, depending on the presence of active transporters. Within the cells, the pH varies greatly between organelles. This may affect drug distribution. In particular, lysosomes (cellular organelles playing a key role in cellular metabolism) may be a site of accumulation for lipophilic and uncharged drugs. Because of the acidic environment within the lysosomes and the pKa of the drugs (see “Drug-specific properties that depend on the environment”), the drugs get positively charged, which makes them more hydrophilic and limits their diffusion back into the brain cells and the brain ECF [82, 83]

#### Drug binding

**Fig. 8:**
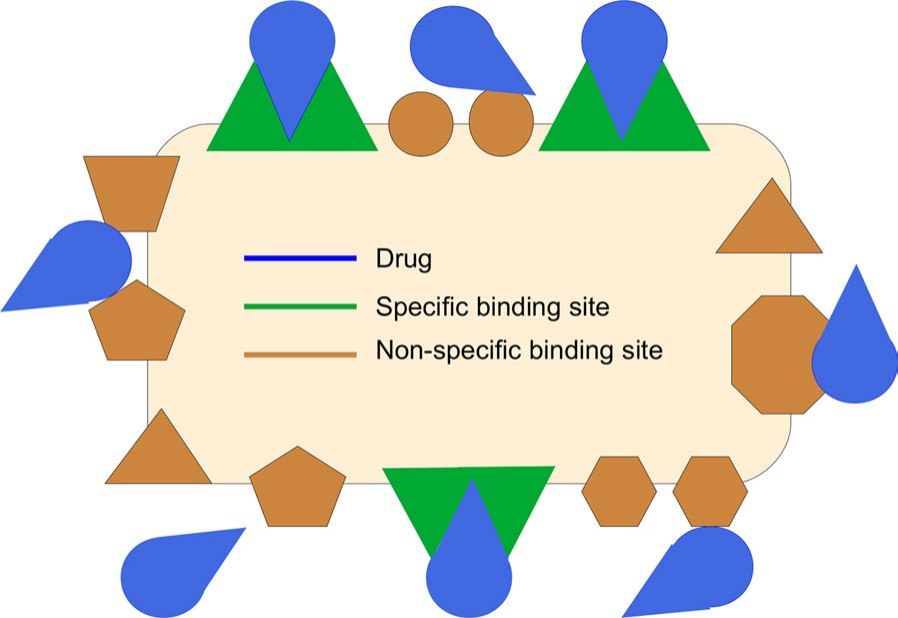
Specific versus non-specific binding. Specific binding involves the (strong) binding of the drug (blue) to the target its intended to bind to (green). Non-specific binding of a drug (blue) to components of the brain (brown) is weaker. However, due to their diverse nature, more non-specific binding sites are present

Drug binding can be classified into specific binding, in which a drug binds to a specific binding site (target), and non-specific binding, when a drug binds to components of the brain (Fig. 8). A target site can be a receptor, enzyme, transport protein or ion channel. Based on their location, drug targets may be classified as extracellular or intracellular, where they may be located within the cytoplasm or the nucleus of a cell. The effect of a drug is directly proportional to the amount of drug bound to its target [84] and a drug only induces its effect during the period it is bound to its target [85, 86]. The interaction of a drug with a non-specific binding site does not result in a (desired) effect. Mostly, non-specific binding involves the interaction of a drug with proteins or other components of the brain that the drug is not intended to bind to. Due to their diverse nature, non-specific binding sites are generally more abundant than targets.

However, drug binding to non-specific binding sites is generally weaker than drug binding to its target. Upon binding, the drug and its binding site form a complex until the drug dissociates to release the drug and the binding site. Drug binding kinetics describe the concentrations of free and bound drug, based on the time a drug interacts with its binding site. This time is known as the drug residence time. The drug residence time is determined by the rates of association and dissociation of a drug to and from its binding site. These, together with the concentration of free drug and the free binding sites, determine the concentrations of free and bound drug (see [87] for a review on this topic). The drug dissociation rate has been thought of as the main determinant of drug-target interaction [87]. However, a recent study shows that the drug association rate can be equally important to determine the duration of drug-target interactions [88].

#### Drug metabolism

Metabolic enzymes (see “Brain-specific properties”) convert active drug to inactive drug. Alternatively, they may transform inactive drug into its active form. Either way, the enzymes affect the concentration of active drug. At the level of the BBB and the BCSFB as well as in the ependymal cells (the cells between the brain ECF and the CSF in the brain ventricles, see Fig. 1b), metabolic enzymes may degrade or inactivate drug and thereby limit the transport of *active* drug across the BBB [45, 65, 89–91]. Within the brain tissue, cytochrome P450 enzymes may be located near drug targets. This may significantly decrease the concentration of active drug and thereby affect drug-target interactions and drug response [46, 92, 93]. The cytochrome P450 metabolic activity on a drug can be affected by competing compounds (compounds that also interact with cytochrome P450), such that co-administration with other drugs or ingestion of certain foods may alter the presence of active drug.

### Factors that may lead to spatial differences in concentration-time profiles of drugs in the brain

Brains are not homogeneous in structure and properties. Therefore, the concentration of a drug within the brain is likely to differ over space. The spatial distribution of a drug is affected by all processes discussed in the previous subsections. Local variations in these processes give rise to local variations in drug distribution. A quantitative understanding is needed on how the various factors of variability affect local drug distribution. Below, we summarise common sources of spatial variability affecting local drug concentration-time profiles within the brain.

#### The brain capillary bed, capillary density and cerebral blood flow

Under normal conditions, the density of the brain capillaries varies within the brain and depends on the local energy needs within the brain [94]. The brain capillary density is higher in grey matter than in white matter due to increased energy demands in grey matter [94–96]. The brain capillary blood flow is also responsive to local brain activity. During stimulation of a functionally active brain area, the corresponding brain arterioles dilate and the blood flow increases in the brain capillaries supplying the area [97, 98]. Both the brain capillary density and the brain capillary blood flow are sensitive to physiological and pathological conditions. Tumours may sprout new blood vessels [99, 100] or may locally reduce blood flow in order to obtain nutrients [100, 101]. Moreover, the brain capillaries may dilate as a response to ischemia (deficiency in blood supply) to increase the influx of oxygen [17, 102–104], while hypertension (high blood pressure) may decrease the number of capillaries [105].

#### Dynamic regulation of BBB functionality

The BBB functionality is responsive to environmental changes. In certain disease conditions, when the BBB is affected, the width of the space between the brain endothelial cells increases due to disruption of the tight junctions. This allows for an increase in paracellular transport, in particular that of larger molecules which normally cannot pass through the intercellular space [8]. This disruption may be local in case of a local disease, such as a local brain tumour. Disruptions of the BBB have most impact on drugs that normally have difficulty crossing the BBB.

#### Diffusion and brain ECF bulk flow

Diffusion in the brain ECF differs between the grey matter and the white matter. In the presence of the neural fiber tracts of the white matter, diffusion is anisotropic (i.e. has a different value when measured in different directions) and depends on the arrangement of the fiber tracts [106]. Hence, while the diffusivity of a compound in the brain ECF of grey matter can be described by one single value, the diffusivity of a compound in the brain ECF of white matter should be described by a tensor containing the diffusivities in all directions [106]. The brain ECF bulk flow can be locally increased, for example as a result of oedema [107]. Oedema is the excessive accumulation of fluid in the intracellular or extracellular space of the brain. It is a common symptom of many brain diseases and may be caused by breakdown of the BBB (see “Dynamic regulation of BBB functionality” above), local brain tumours, and altered metabolism.

#### Intra-/extracellular exchange

The cellular parts that make up the brain tissue differ between the white matter and the grey matter (see also “The brain tissue and the CSF” in “Brain-specific properties”). While the white matter contains few cell bodies and many axons, the grey matter contains many cell bodies and few axons. The white matter consists of the myelinated axons of neurons, glial cells, and brain capillaries. In contrast, the neuronal cell bodies and dendrites make up most of the grey matter in the superficial part of the brain. Not only cell types, but also cell densities have been found to differ per brain region in monkeys [108]. Finally, the concentration of binding sites can differ per cell and cell type, depending on the drug and the target it is aiming for.

#### Binding

The location of drug targets is crucial, as a drug needs to be able to distribute to this site in order to be effective. In case of local disease, the drug target area, such as a brain tumour, commonly has different physiological properties than the rest of the brain. This affects the drug distribution to its target.

#### Brain metabolism

The expression of metabolic enzymes may differ locally. A recent study has demonstrated that the spatial distribution of two brain metabolic enzymes, glutamine synthetase and glycogen phosphorylase, is not homogeneous in honeybee brains and differs between as well as within regions [109].

## Existing models on the local distribution of drugs in the brain

Understanding how a drug distributes into and within the brain is crucial to accurately predict the effect of a drug that targets the brain. However, much is still unknown about drug distribution within the brain. Mathematical modelling can provide information that is otherwise hard or impossible to obtain by experiments only. Thereby, models help to gain insight into the mechanisms under study. In the next subsections, existing models on (processes related to) drug distribution in the brain are reviewed. In the first two subsections, models on drug transport through the brain capillary system (“Modelling drug transport through the brain capillary system”) and across the BBB (“Modelling drug transport across the BBB”) are described. The next four subsections describe models on the drug distribution within and elimination out of the brain, including drug distribution within the brain ECF (“Modelling drug transport within the brain ECF”), intra-extracellular exchange (“Modelling intra-extracellular exchange”), drug binding kinetics (“Modelling drug binding kinetics”) and drug metabolism (“Modelling drug metabolism in the brain”). Ranges of values and units for the parameters that are relevant for each process are given for rat and human in the Appendix. Models on the exchange between several compartments representing parts of the brain or states of the drug are covered in “Modelling drug exchange between compartments”. A summary is given in Table 2. Finally, in “Integration of model properties”, an overview is given on the current state of the art of models on drug distribution within the brain that integrate mathematical descriptions of drug distribution within the brain. Of this, a summary is given in Table 3.

### Modelling drug transport through the brain capillary system

**Fig. 9:**
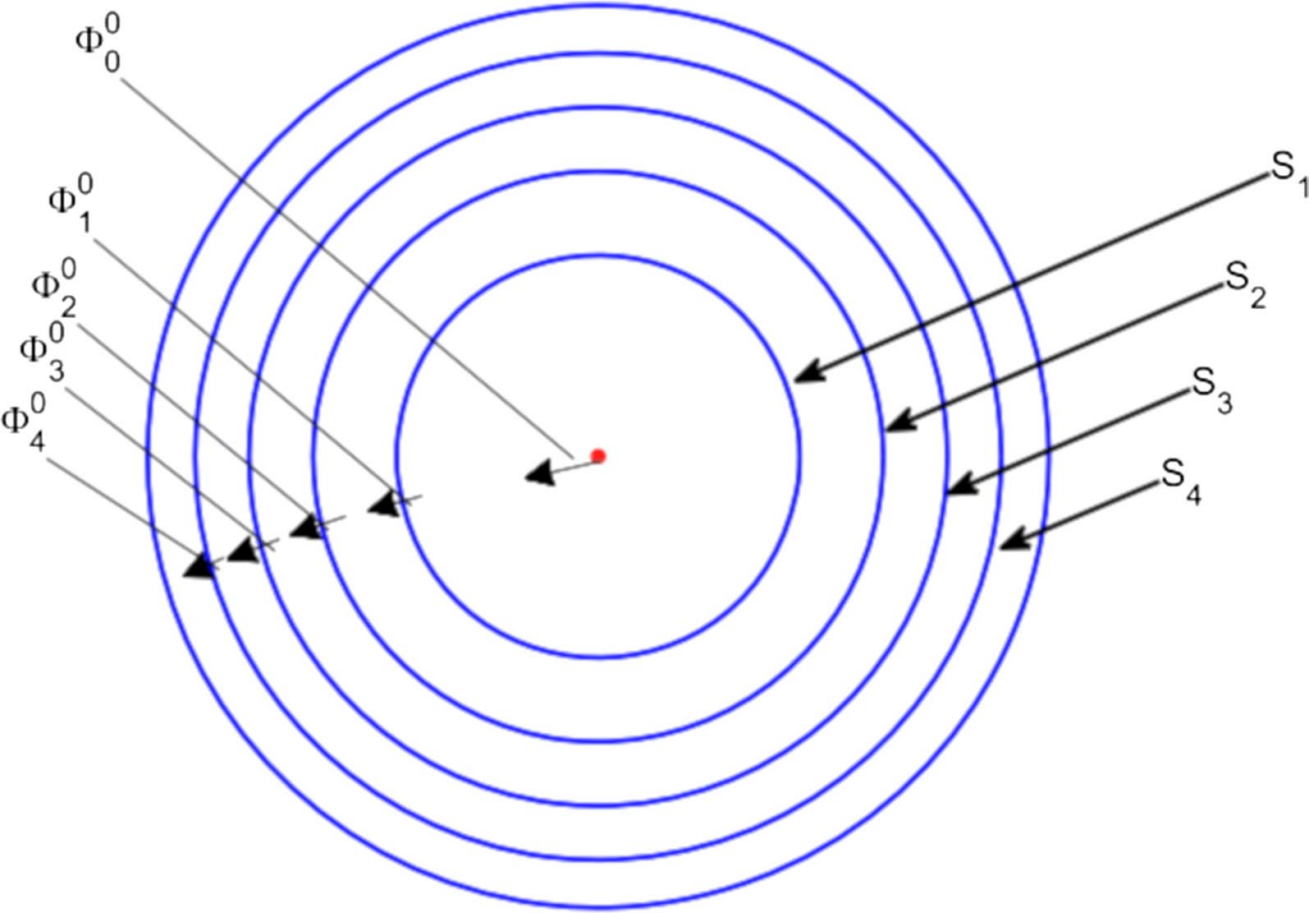
Circular representation of the Krogh cylinder. A brain capillary (red point in the middle) is surrounded by four layers of brain tissue, represented by subunits *S*_j_($1\le {\text{j}}\le 4$) (blue). Here $\Phi$_0_ describes the exchange rate through the BBB and $\Phi$_j_($1\le {\text{j}}\le 4$) describes the drug diffusion flux between the brain tissue subunits. Adapted with permission from [111]

We only mention models that specifically focus on drug transport into the brain and thereby on drug delivery by the brain capillary network. The distribution of compounds from the capillaries into a tissue, such as the brain, can be represented by a Krogh cylinder. A Krogh cylinder represents the tissue as a cylinder with a single capillary at its centre [110]. The model is well established and has been extensively used to describe the supply of oxygen and other molecules to a wide range of tissues, including the brain [111]. An example of a Krogh cylinder is given in Fig. 9, where a brain capillary is surrounded by layers of brain tissue [111]. There, the brain is represented by four subunits, denoted by *S*_j_(1$\le$ j $\le$ 4). Drug diffusion fluxes occur between the brain capillary and the brain tissue, denoted by $\Phi$_0_, and between the brain tissue subunits, denoted by $\Phi$_j_(1$\le$ j $\le$ 4). The Krogh cylinder can be used to determine the effect of the brain capillary blood flow on simple passive drug transport across the BBB. The rate constant of passive drug transport into the brain, *k*_in_, can be related to rate of the brain capillary blood flow, *Q*, and the fraction of compound extracted into the brain, *E*, by the Renkin–Crone equation [112, 113]:

1 $$\begin{aligned} \begin{aligned} k_{\mathrm {in}}&= \frac{QE}{V_{\mathrm {brain}}},\\ \text {with } E&= 1-\mathrm {e}^{\frac{-PS}{Q}} \\ \text { or } E&= \frac{C_{\mathrm {in}}-C_{\mathrm {out}}}{C_{\mathrm {in}}}, \end{aligned} \end{aligned}$$

with *V*_brain_ (L) the volume of the brain, *E* the compound extraction ratio, *PS* (m s^−1^ m^2^) the BBB permeability surface area product, *C*_in_ (mol L^−1^) the concentration of drug entering the brain capillary and *C*_out_ (mol L^−1^) the concentration of drug leaving the brain capillary. From Eq. (1), it follows that for a drug that readily crosses the BBB (*PS* is high), drug extraction from the blood plasma into the brain ECF is limited by the brain capillary blood flow rate. If a drug has difficulties crossing the BBB, (*PS* is low), drug extraction from the blood plasma into the brain ECF is limited by the permeability of the BBB.

**Fig. 10:**
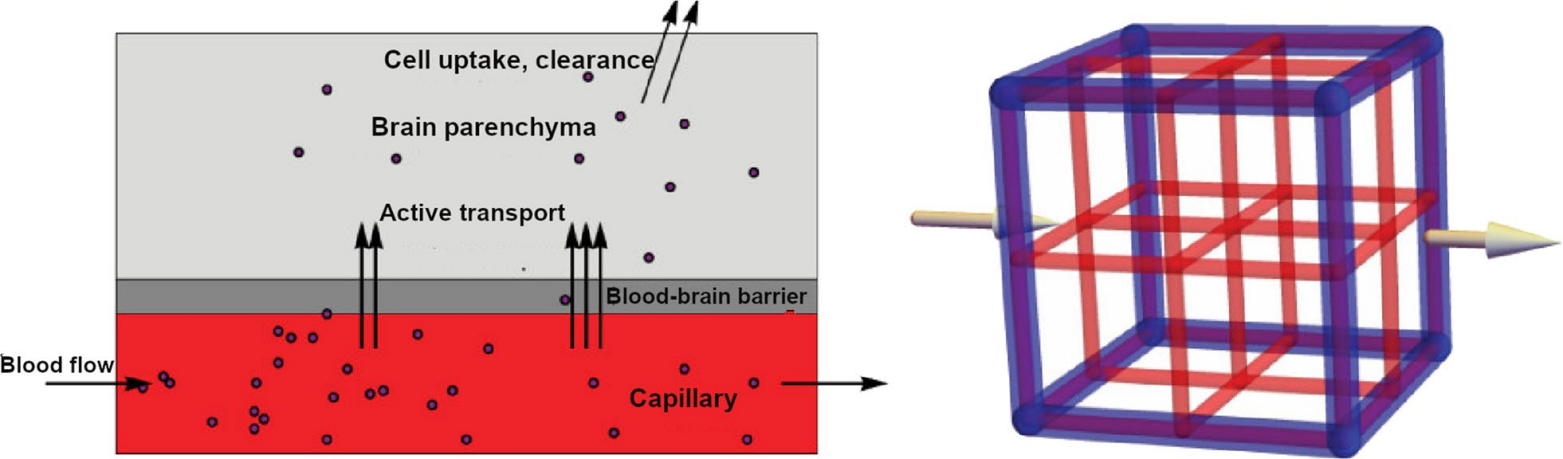
Model on drug transport within the brain capillaries, active drug transport across the BBB and subsequent metabolism [125]. Spatial differences within the brain are, however, *not* considered. Left: a simplified model for active drug transport within the capillaries, across the BBB and subsequent metabolism [125]. The black circles represent the drug. Right: a schematic depiction of the lattice refinement process [125]. A cubic lattice (blue) represents a piece of brain tissue with a volume of 1 cm^3^. The cubic lattice can be replaced by a network of smaller cubic lattices (red). The larger blue unit and the multiple smaller units fill out the same computational volume. The arrow indicates the direction of the blood flow through the large unit. Both images by [125] are licensed under CC BY 4.0

The Krogh cylinder is limited to a single segment and does not take diffusion along the barrier into account. It drives on the assumption that *PS* is a physiological constant, while in fact, it is not identical to the physiological permeability as it highly depends on brain capillary blood flow rate and radius [114]. Recently, large-scale anatomical models of brain vascular networks have been developed. There, entire brain vascular networks are constructed based on segmentation of medical images [115–119] or geometric construction [120–124]. These networks consist of a multitude of blood vessel segments connected by nodes, where parameters defining the network (such as blood vessel radius, volume and length) are based on images, experimental data or random distribution. These brain vascular networks can be applied to drug delivery [116, 125]. In a model on drug delivery to brain tumours, an image-based brain capillary network is coupled to a cubic mesh representation of the brain tissue [116]. There, a system of differential equations describes drug transport within the blood vessels, (passive) drug transport to the tissue and drug diffusion and decay within the tissue. A recent mathematical model describes the drug delivery to the brain by the brain capillaries and subsequent active transport across the BBB [125] (Fig. 10, left). In the network, each brain capillary supplies its own volume of brain tissue. The authors do not consider passive transport across the BBB. In this model, a network of brain capillaries is described with a constant topology of cubic lattices. The total network of cubic lattices represents a piece of brain tissue with a volume of 1 cm^3^. The volumes of the brain tissue lattices in the network are identical and spatial differences within the brain are not considered. A constant concentration of drug enters the network at the left surface (x = 0). The overall blood flow is directed from the left to the right side of the network (from x = 0 to x = 1), see Fig. 10 (right).

While the *rate* of drug transport across the BBB is affected by the BBB permeability and the rate of brain capillary blood flow, the *amount* of drug that crosses the BBB is affected by drug binding to blood plasma proteins. Drug binding to blood plasma proteins reduces the concentration of unbound drug that is able to cross the brain. However, only few modelling studies take drug binding to blood plasma proteins into account. In one example, the high affinity of a chemotherapy drug, Doxorubicin, for blood plasma proteins is described by partitioning the concentration of drug into free and plasma protein-bound drug [126].

### Modelling drug transport across the BBB

Most drugs enter the brain from the blood and therefore have to cross the BBB. Therefore, it is important to include the BBB in models on drug distribution within the brain. Passive and active transport across the BBB require different modelling approaches. These are described below.

#### Passive BBB transport

**Fig. 11:**
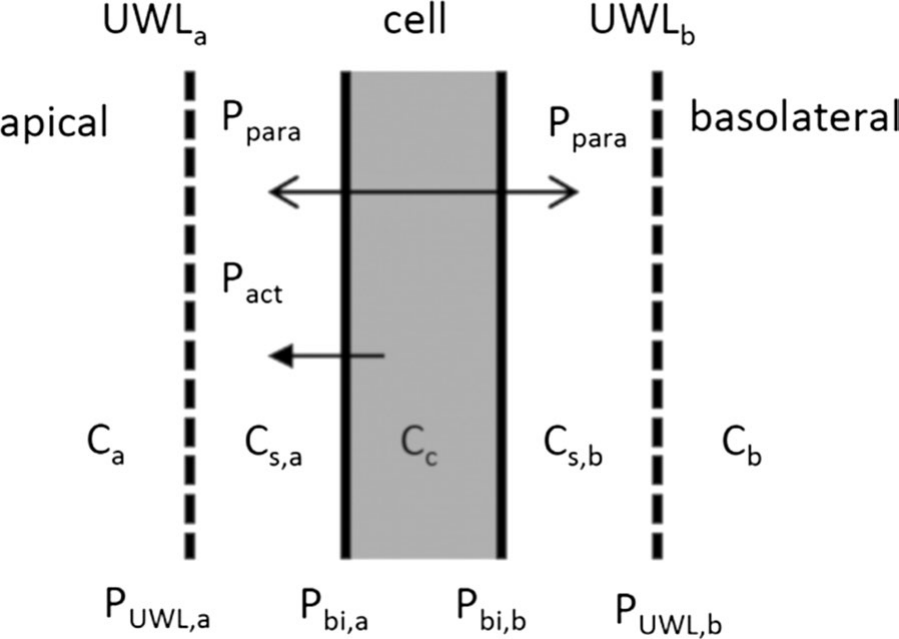
Schematic representation of the model by Trapa [54] discussed in the text. In the model, used to extract active permeability from in vitro transwell permeability experiments, passive (*P*_pas_) and active (*P*_act_) permeability, paracellular (*P*_para_) transport, and the effects of unstirred water layers (UWLs) are considered on both the apical and basolateral sides of the membrane. Adapted with permission from [54]

Drug transport across the BBB is often described as a loss of compound from the brain ECF, i.e. the unidirectional and irreversible transport of drug from the brain ECF to the blood plasma [107, 127–132]. However, passive transport across the BBB is bidirectional: drug is transported from the blood to the brain ECF and from the brain ECF to the blood. Below, several methods of quantification of passive BBB transport are discussed. The passive flux of drug across the BBB between the blood plasma and the brain ECF, $\Phi$, is bidirectional and perpendicular to the BBB. It depends on the BBB permeability and on the drug concentration difference between the blood plasma and the brain ECF. It can be defined as follows [111, 126, 133]:

2 $$\begin{aligned} \Phi _{\mathrm {pas}}= P(C_{\mathrm {pl}}-C_{\mathrm {ECF}}), \end{aligned}$$

with $\Phi$_pas_ (mol m^−2^ s^−1^) the bidirectional passive flow rate of drug per unit area of the BBB, *P* (m s^−1^) the permeability of the BBB to the drug, *C*_pl_ (mol m^−3^) the concentration of drug in the blood plasma and *C*_ECF_ (mol m^−3^) the concentration of drug in the brain ECF. The change in drug concentration in the brain ECF as a consequence of bidirectional, simple passive drug transport across the BBB can be described using a rate constant [129, 132, 134–136] or transfer clearance parameter [137–142]:

3 $$\begin{aligned} \begin{aligned} \frac{\mathrm {d}C_{\mathrm {ECF}}}{\mathrm {dt}}&= k_{\mathrm {BBB}}(C_{\mathrm {pl}}-C_{\mathrm {ECF}}) [1] \text { or} \\ V_{\mathrm {ECF}} \frac{\mathrm {d}C_{\mathrm {ECF}}}{\mathrm {dt}}&= CL_{\mathrm {BBB}}(C_{\mathrm {pl}}-C_{\mathrm {ECF}}),\\ \text {with } C_{\mathrm {ECF}}&= \frac{A_{\mathrm {ECF}}}{V_{\mathrm {ECF}}}, \end{aligned} \end{aligned}$$

where *k*_BBB_ (s^−1^) is the rate constant of drug transport across the BBB, *CL*_BBB_ (m^3^ s^−1^) is the transfer clearance of drug transport across the BBB, *A*_ECF_ (mol) is the molar amount of drug in the brain ECF and *V*_ECF_ (m^3^) is the volume of the brain ECF. In some studies the amount of drug in the brain *tissue* (including the brain ECF and the brain ICF) is modelled rather than the amount of drug in the brain ECF, i.e. *A*_brain_ is used rather than *A*_ECF_ [134, 136]. The passive flux of drug across the BBB, defined by Eq. (2), is the sum of the passive flux due to transcellular transport and the passive flux due to passive paracellular transport. Therefore, the passive permeability, *P*, can be given by [143]

4 $$\begin{aligned} P = P_{\mathrm {trans}}+ \frac{D_{\mathrm {para}}}{W_{\mathrm {TJ}}} \end{aligned},$$

where *P*_trans_ (m s^−1^) is the passive transcellular permeability, *D*_para_(m^2^ s^−1^) is the diffusivity of a drug through the BBB intercellular space and *W*_TJ_ (m) is the width of the tight junction. Equation (4) is based on the assumption that the width of the tight junction equals the distance travelled by the diffusing drug through the BBB intercellular space. However, electron microscopy shows that the BBB tight junctions have a tortuous shape [144] and therefore *W*_TJ_ likely underestimates the actual distance travelled by the diffusing drug. Paracellular diffusion only occurs at $0.006\%$ of the total surface area of the BBB [143]. Therefore, correction factors (BBB surface area fractions) need to be used that take into account the relative contributions of passive paracellular and passive transcellular transport [143]. Transport through unstirred water layers on both sides of the BBB is included in a recent model that extensively describes compound transport across both the apical (blood-facing) and abluminal membranes (brain-facing) of the cells of the BBB [54]. On both sides of the membrane, the effects of passive transcellular permeability, paracellular transport, active permeability and unstirred waterlayers on compound concentrations within the BBB, brain ECF and unstirred water layers are described (Fig. 11).

#### Active BBB transport

Active transport involves the movement of a molecule from one side to the other side of the membrane against the concentration gradient and therefore, it requires energy. Active transport is unidirectional and mediated by active transport proteins and requires other descriptions than those for passive transport. Below, several methods of quantification of active BBB transport are discussed. In its most simple form, the total flux ($\Phi$_tot_) across the BBB by both passive and active transport is described in the same manner as the passive permeability (Eq. (2)), thereby ignoring the unidirectionality of active transport and saturation of active transport proteins:

5 $$\begin{aligned} \begin{aligned} \Phi _{\mathrm {tot}}&= P_{\mathrm {tot}}(C_{\mathrm {pl}}-C_{\mathrm {ECF}}) \\ \text {or } \Phi _{\mathrm {tot}}&= P AF_{\mathrm {in}}(C_{\mathrm {pl}})-P AF_{\mathrm {out}}(C_{\mathrm {ECF}}), \end{aligned} \end{aligned}$$

where *P*_tot_ (s^−1^) is the rate of total (passive + active) transport) across the BBB, *AF*_in_ is the affinity of a drug to active transport into the brain [141] and *AF*_out_ is the affinity of a drug to active transport out of the brain [141].

The total permeability, *P*_tot_, is often described as the product of the passive BBB permeability *P* multiplied by the blood–brain partition coefficient [1, 138, 139, 141, 142, 145]. Alternatively, active transport of drug out of the BBB can be described by an active permeability, *P*_act_ [54]. This active permeability, *P*_act_ can be specific for particular transporters, such that *P*_act_ equals the sum of active BBB transport by individual transporters, including *P*_P-gp_ and *P*_BCRP_ for P-gp and BCRP (see “Drug transport across the brain barriers”) [54]. The descriptions of the total flux, $\Phi$_tot_, in Eq. (5) are not valid in the presence of paracellular transport, because in that case the compound circumvents the cells and does not interact with active transporters on the cells [54]. Then, Eq. (2) should be used. Active transport is commonly assumed to work according to Michaelis-Menten kinetics, which are originally used to describe enzyme conversion. In this way, the active clearance, *CL*_act_, of drug across the BBB into or out of the brain is modelled as follows [1, 127, 138, 139, 146, 147]:

6 $$\begin{aligned} CL_{\mathrm {act}} =\frac{T_{\mathrm {m}}}{K_{\mathrm {m}}+C} \end{aligned},$$

with *T*_m_ ($\upmu \text {mol}\;\text {L}^{-1}\;{\text{s}}^{-1}$) the maximum rate of drug transport across the BBB (negative for outward transport), *K*_m_ ($\upmu \text {mol}\;\text {L}^{-1}$) the concentration of free drug at which half of *T*_m_ is reached and *C* ($\upmu \text {mol}\;\text {L}^{-1}$) the concentration of drug in the blood plasma, *C*_pl_ (in case of active inward transport) or in the brain ECF, *C*_ECF_ (in case of active outward transport).

### Modelling drug transport within the brain ECF

On a microscopic scale, diffusion of a compound can be described by a random walk of the molecules and on a macroscopic scale this translates to the diffusion equation [148]. This equation describes the distribution of a compound through a medium, such as the brain ECF:

7 $$\begin{aligned} \frac{\partial C}{\partial t} = D \nabla ^2 C, \end{aligned}$$

with *D* the diffusion coefficient ($\text {m}^{2}\;\text{s}^{-1}$) and *C* the concentration of the compound in the medium ($\text {mol}\;\text {L}^{-1}$).

Within the brain ECF, diffusion of molecules is reduced by the hindrance of obstacles, including cells (see “Drug transport within the brain fluids” in “Processes affecting drug distribution within the brain”). To take the complexity of the brain ECF into account, the diffusion equation should be modified by including the tortuosity ($\lambda$) and brain ECF volume fraction ($\alpha$) [16, 149]. Here, $\lambda$ describes the hindrance posed on diffusion by a geometrically complex medium, such as the brain ECF, in comparison to a medium without obstacles, such as water [150]. The parameter $\alpha$ is the ratio of the volume of the brain extracellular space to the volume of the total brain tissue. As indicated before, the distribution of a drug within the brain is also affected by exchange with the brain capillaries (see “Modelling drug transport through the brain capillary system” and “Modelling drug transport across the BBB”), the brain ECF bulk flow, intra-extracellular exchange (see “Modelling intra-extracellular exchange”), drug binding (see “Modelling drug binding kinetics”) and drug metabolism (see “Modelling drug metabolism in the brain”). Charles Nicholson has done a considerable amount of work to accurately describe the distribution of a drug within the brain ECF and the processes that affect it. One of the main results of his work is a modified diffusion equation that describes the distribution of a drug within the brain ECF [151]. There, he assumes that the drug is administered directly to the brain. The entry of a compound from the blood across the BBB into the brain ECF is not taken into account. The modified diffusion equation is widely used to investigate drug distribution within the brain ECF [127, 128, 152, 153]. and is as follows:

8 $$\begin{aligned} \frac{\partial C}{\partial t} = D^*\nabla ^2 C + \frac{Q}{\alpha } - v \nabla C - k'C - \frac{f(C)}{\alpha }, \end{aligned}$$

with *C* the concentration of drug within the brain ECF, $\alpha = \frac{V_{\mathrm {ECF}}}{V_{\mathrm {tissue}}}$ and $D^* = \frac{D}{\lambda ^2}$, with $\lambda = \sqrt{\frac{D}{D*}}$.

The first term describes the diffusion of a compound with an effective diffusion coefficient D* (m^2^ s^−1^). This is the normal diffusion coefficient (D) corrected by the tortuosity, $\lambda$, describing the hindrance by cells imposed on diffusion of the compound within the brain ECF (see “Drug transport within the brain fluids”). The second term is a source term, where *Q* (mol L^−1^ s^−1^) describes the local release of substances within the tissue, e.g. by injection or infusion. The factor $\alpha$ corrects for the fact that drug is released into the brain tissue but distributes only within the brain ECF. The third term describes the transport of a compound by brain ECF bulk flow, where *v* (m s^−1^), is the bulk flow velocity of the brain ECF. The fourth term includes *k*′, which is a first order elimination rate constant that describes the permanent loss of a compound into the cells or into the blood. Finally, *f(C)* (mol L^−1^ s^−1^) describes the binding of molecules to the extracellular matrix, specific receptors or transporters. This term, however, does not include drug binding kinetics and does not distinguish between specific and non-specific binding. Again, the factor $\alpha$ corrects for the fact that drug resides in the brain ECF only.

#### Modelling cells in the modified diffusion equation

**Fig. 12:**
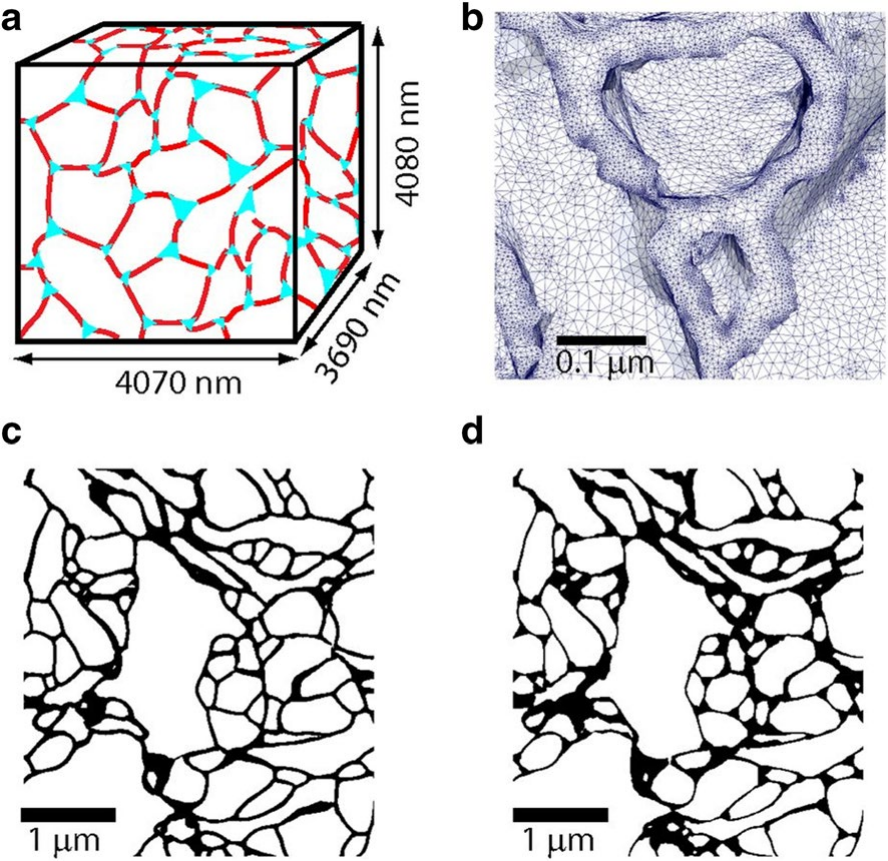
Model systems and microscopic structure of the brain extracellular space as formulated in [38]. **a** Schematic representation of the reconstruction of the brain extracellular space generated by electron microscopy. Sheets (the spaces between two adjacent cells) are in red, while tunnels (the spaces at the junctions of three or more cells) are in cyan. **b** Close-up of the electron microscopy reconstruction showing typical sizes of the 84 million tetrahedrons used in the simulation. **c**, **d** Electron microscopy reconstruction of the brain extracellular space by Kinney et al. [157] with a small tunnel volume fraction (**c**) and with a larger tunnel volume fraction (**d**). Adapted with permission from [38]

Cells are the major hindrance to movement by diffusion within the brain ECF. However, by default the modified diffusion equation (8) only implicitly includes cells by taking their hindrance into account by the tortuosity. In other models on drug distribution within the brain, brain cells are commonly represented as one compartment. There, drug can be exchanged between the cellular compartment and the extracellular compartment [106, 126, 132, 140, 154, 155] (see “Modelling drug exchange between compartments”). However, both the tortuosity and the compartmental representation of cells are simplifications of a more complex geometry. To more realistically represent transport within the brain ECF, other models explicitly describe cells and their shape [33, 71, 131, 150, 156]. In a recent study on solute transport within the brain ECF, the cells in the brain ECF are modelled as Voronoi cells (cells of which the boundaries are determined by the distance between the cell center and the center of other cells) to represent the heterogeneity of brain cells [33]. Moreover, a three-dimensional representation of the brain neuropil (i.e. the brain grey matter) together with its brain ECF allows for a realistic representation of the brain extracellular space and transport within the brain ECF [38, 131, 157]. These studies use ‘sheets and tunnels’ to represent the brain ECF. There, ‘sheets’ represent the small space between two adjacent cells, while ‘tunnels’ represent the space at the junction of three or more cells [38] (Fig. 12).

#### Determining the parameters

The values of parameters in the modified diffusion equation, such as $\lambda$ (tortuosity) and $\alpha$ (brain ECF volume fraction), can be obtained by experimental techniques, computational simulations and theoretical calculations. Experimental techniques include dual-probe microdialysis, integrative optical imaging, real-time iontophoresis, tracer-based magnetic-resonance imaging (MRI) and diffusion tensor imaging. The characteristics of the experimental techniques are summarised in Table 1. All of the techniques except diffusion tensor imaging are invasive and therefore usually performed in the rat. Diffusion tensor imaging is non-invasive and provides information about diffusion within the human brain. The techniques provide information on drug transport within the brain ECF, such as the geometry of the brain extracellular space and local drug concentrations at different points in space. From this, parameter values related to diffusion, including $\lambda$, $\alpha$ and the diffusion tensor (measuring the diffusivity in several directions) can be calculated. Computational methods are used to estimate values of coefficients for the modified diffusion equation. When experimental data do not suffice, the remaining parameters can be estimated based on a fit with experimental data. This is often done in pharmacokinetic models (see “Modelling drug exchange between compartments”. In addition, more rigorous methods exist for determining parameter values of drug transport within the brain, as is explained in [158]. To estimate parameter values without any experimental data, Monte Carlo simulations are commonly used. These are predictive simulations in which molecules perform a random walk in a pre-set geometry of the brain extracellular space to mimic molecular diffusion [69, 150, 159, 160]. These simulations then give information on how the geometry of the brain extracellular space (see “Drug transport within the brain fluids”) affects diffusion of a drug within the brain ECF. Theoretical calculations can be used to calculate diffusion in the brain ECF and the brain ECF bulk flow velocity. Diffusion in the brain ECF compared to diffusion in a cell-free medium is quantified by the tortuosity. The tortuosity is calculated based on the effect of the presence and geometrical arrangement of cells on diffusion. This effect is measured either as the increase in distance travelled by the diffusing drug [161–163] or as the increase in time needed for the diffusing drug to travel from point A to point B [71, 150]. The brain ECF bulk flow velocity is commonly determined from the fluid velocity field computed with the Navier-Stokes equations (a set of partial differential equations describing the movement of fluid) [33] or with equations using the pressure of the brain ECF and the hydraulic conductivity (the ease with which a fluid can move through a porous medium like the brain extracellular space) [132, 164].

Model input can also be derived from subject-specific data [130, 131, 165–167]. In a recent study, the brain matter was reconstructed from MRI-images using open source software in order to model protein transport within the brain tissue with a basic reaction-diffusion equation [167].

**Table 1:** Experimental techniques to determine parameter values for the diffusion equation

Technique	Explanation	References
Dual-probe microdialysis	A probe measures local drug concentration after diffusion from the first (release) probe	[128, 152]
Integrative optical imaging	Microscopical imaging of macromolecule attached to fluorescent marker. Uses the hypothesis of restricted diffusion^a^	[72, 76, 156, 160, 168–174]
Real-time iontophoresis	Changes in electrical potential induced by charged ions are recorded	[149, 175]
Tracer-based MRI^b^	Magnetic sensitive contrast agents are attached to water molecules and imaged	[30, 176]
Diffusion MRI/diffusion tensor imaging	Non-invasive techniques to study the random movement of molecules and obtain the diffusion tensor^c^	[130, 131, 166, 177]

^a^This states that with increasing molecule size, diffusion becomes less as the molecules approach the width of the brain extracellular space [178, 179]

^b^Currently the only measurement to provide a three-dimensional visualization of the brain ECF bulk flow [30, 176]

^c^The diffusion tensor measures the diffusivity in several directions, thereby assessing tissue anisotropy

#### Applications of the modified diffusion equation

The modified diffusion equation (8) can be adjusted according to the specific purpose of a study. For example, to take bidirectional BBB transport into account, one or two rate constants (s^−1^) can be included that specifically quantify the concentration-dependent exchange between the blood plasma and the brain ECF in one or two directions [128, 133, 135, 180, 181]. To account for the anisotropic diffusion within the white matter, a diffusion tensor can also be used instead of the effective diffusivity D* [182]. The modified diffusion equation has proven useful in predicting the local distribution profile of a drug after several invasive experimental measurements, such as microdialysis [128, 135, 152, 181, 183–188]. Using the diffusion equation to predict the impact of invasive experimental techniques on the local distribution of a drug helps to evaluate experimental measurements. For instance, predictive studies have suggested that microdialysis increases $\alpha$ and thereby may affect the spatial drug distribution profile of a substance [135, 181]. The predictions of diffusion equation may be used to better target local drug delivery. For example, a mathematical model containing the diffusion equation is developed to aid the optimization of treatments by controlled release polymer implants [153]. There, optimization refers to a trade-off between effective drug distribution within the brain and minimization of side effects [153]. Other examples using Eq. (8) include studies on the local distribution of a drug within the rat [153, 180, 189–191], dog [133], pig [192] and human [166, 192, 193] brain around the site of application after direct administration of drug into the brain ECF through polymer implants [153, 189], perfusion [133, 180, 185, 192] or convection-enhanced delivery (the delivery of drug by a pressure gradient directly to the brain ECF, thereby bypassing the BBB) [166, 185, 190–193].

#### Modelling drug transport within the CSF

Modelling drug transport within the CSF has great similarities to modelling drug transport within the brain ECF. Yet for the sake of completeness, drug transport within the CSF will be shortly discussed below. Drug transport within the CSF is generally described with an advection–diffusion equation [194, 195]:

9 $$\begin{aligned} \frac{\partial \mathrm {C}}{\partial \mathrm {t}} = D \nabla ^2 C - v \nabla C, \end{aligned}$$

with *C* the concentration of drug within the CSF, *D* the diffusion of drug within the CSF (note that hindrance by the cells imposed on diffusion is not taken into account as there are no cells in the CSF), and *v* the CSF bulk flow. It is clear that Eq. (9) is very similar to Eq. (8). The equation has been applied to study the effect of drug-specific and system-specific properties on drug distribution within the CSF. In one recent study, the equation is used to predict drug distribution in patient-specific CSF and with these predictions improve CSF drug delivery [196]. In another recent study, a model is proposed to study the transport of a solute within the CSF [195]. There, a solute refers to drug that is dissolved into the CSF. The solute diffusivity is measured by the Schmidt number, that relates the diffusivity of the drug to the viscosity of the drug-carrying fluid. Due to the ‘slender’ morphology of the spine, that has a length much larger than its diameter and width, diffusion is assumed to be uni-directional. In addition, the brain CSF bulk flow is modelled as a time-averaged Lagrangian velocity (see also [197] for more information).

Data on CSF flow, in some cases subject-specific [198–200] can be provided by mathematical models of CSF dynamics [198–202]. The models can be coupled to models on drug distribution within the CSF, e.g. by providing data on the CSF bulk flow velocity.

### Modelling intra-extracellular exchange

Modelling drug transport across the membranes of the brain cells is analogous to modelling drug transport across the brain barriers (discussed in “Modelling drug transport across the BBB”). In its simplest form, intra-extracellular exchange is quantified by a rate constant, *k’*, that describes the linear uptake of drug into cells [compare to the fourth term of Eq. (7)] [129, 203]. This rate constant is usually multiplied by the brain ECF volume fraction, $\alpha$, to account for the space the cells occupy within the brain:

$$\begin{aligned} f(C) = -\alpha k' C. \end{aligned}$$

However, like BBB transport (discussed in “Modelling drug transport across the BBB”), transport across the cell membrane is bidirectional and passive transport across the cell membrane is driven by the concentration gradient between the brain ECF and the brain ICF. The passive flux of drug across cell membranes between the brain ECF and the brain ICF is therefore analogous to Eq. (2). Analogous to Eq. (3), the change in drug concentration within the cells of the brain can be described as [126, 135, 140, 141, 143, 204]:

$$\begin{aligned} \begin{aligned} \frac{\mathrm {d}C_{\mathrm {ICF}}}{\mathrm {dt}}&= k_{\mathrm {cell}}(C_{\mathrm {ECF}}-C_{\mathrm {ICF}})\ \text { or} \\ V_{\mathrm {ICF}}\frac{\mathrm {d}C_{\mathrm {ICF}}}{\mathrm {dt}}&= CL_{\mathrm {cell}}(C_{\mathrm {ECF}}-C_{\mathrm {ICF}}),\\ \text {with } C_{\mathrm {ICF}}&= \frac{A_{\mathrm {ICF}}}{V_{\mathrm {ICF}}}, \end{aligned} \end{aligned}$$

where *C*_ICF_ ($\upmu \text {mol}\;\text {L}^{-1}$) is the concentration of drug within the brain ICF, *k*_cell_ (s^−1^) is the rate constant of drug transport across the cell membrane, *CL*_cell_ is (L s^−1^) the transfer clearance of drug across the cell membrane and *V*_ICF_(L) is the apparent volume of drug distribution in the brain ICF. Alternatively, the cell membrane permeability surface area product *PS*_cell_ (m^3^ s^−1^) can be used instead of *CL*_cell_ [142]. Active, saturable, transport into or out of the cells is, like active BBB transport (discussed in “Drug transport across the brain barriers”), usually described by Michaelis–Menten kinetics [111, 127, 205] [see also Eq. (6)]:

10 $$\begin{aligned} CL_{\mathrm {act-cell}} = \frac{T_{\mathrm {m-cell}}}{\alpha (K_{\mathrm {m-cell}}+C)}, \end{aligned}$$

where *CL*_act-cell_ is the active transfer clearance of free drug across the cell membrane between the brain ECF and brain ICF, *C* is the concentration of drug within the brain ECF or within the brain ICF, *T*_m-cell_ represents the maximal velocity of the transporter (negative for outward transport) and *K*_m-cell_ is the Michaelis–Menten constant, which is generally assumed to represent the rate of dissociation of drug from its binding sites on the cellular membrane [203, 205].

### Modelling drug binding kinetics

**Fig. 13:**
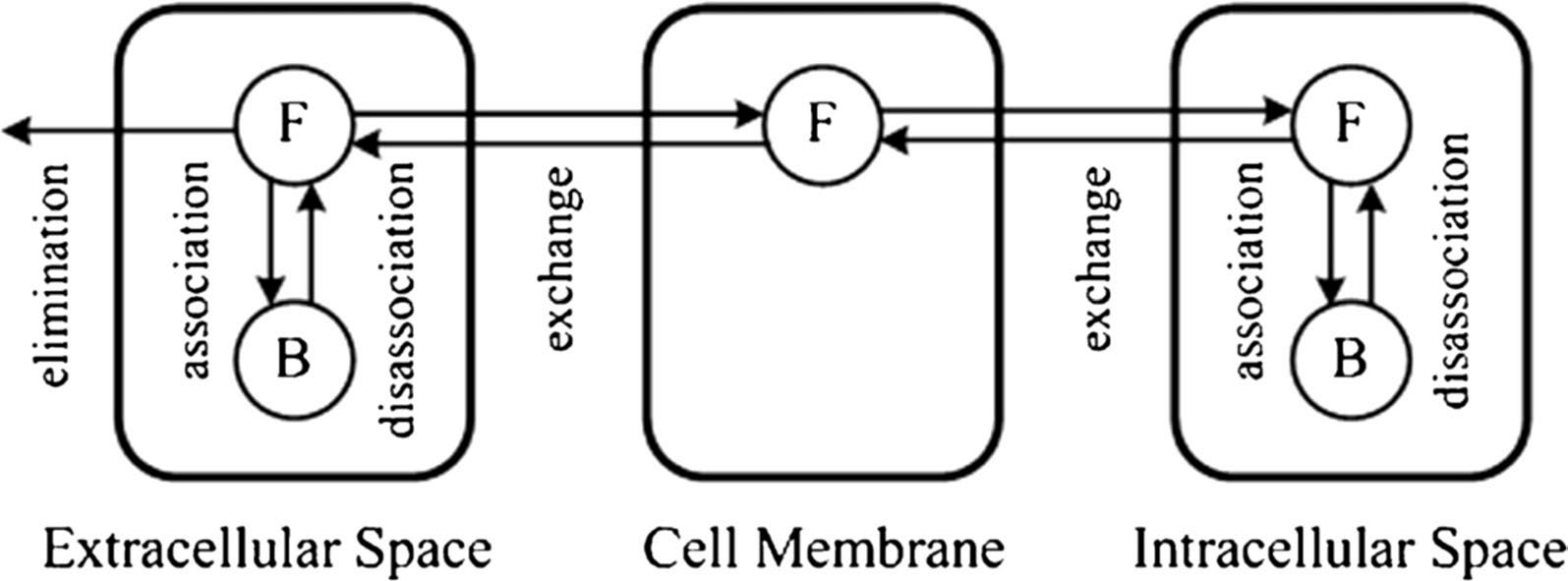
Schematic representation of a model on drug transport in brain tumour and surrounding normal tissue after treatment with convection-enhanced delivery [132]. Both the tumour and the surrounding normal brain tissue consist of three compartments: the extracellular space, the cell membrane and the intracellular space. F and B represent free and protein-bound drug, respectively. Free drug in the brain extracellular space distributes between the tumour and the surrounding tissue by diffusion and brain ECF bulk flow. Within each region, only free drug is available for transport across the cell membrane to the extracellular and intracellular space. Adapted with permission from [132]

Drug binding within the brain is most commonly modelled by a rate constant, that describes the loss of free compound to binding sites [54, 127, 129–131, 140, 142, 145, 153, 185]. However, the *kinetics* of drug binding, which include the rates of association and dissociation of a drug to its binding sites are not considered. Also the concentration-time profiles of free and bound drug are not considered. In a recent study on drug distribution within brain tumours after administration by convection-enhanced delivery, the association and dissociation rates of drug interaction with binding sites in the brain ECF and brain ICF of brain tumours and surrounding tissue are described [132] (Fig. 13). There, free drug in the brain extracellular space distributes between the tumour and surrounding normal tissue by diffusion and bulk flow. Both tumour and normal tissue consists of three compartments: the brain extracellular space, the cell membrane and the intracellular space. Only free drug can cross the membrane to exchange between compartments. Within the extracellular and intracellular space drug binds with proteins. However, this study does not distinguish between binding association and dissociation rate constants, nor does it consider the concentration of binding sites and possible saturation thereof [132]. Models describing concentration changes in free drug *and* free binding sites do exist for other areas of the body than the brain. A recent work on the local delivery of drug to the arterial wall describes concentration changes of free and bound drug in the (non-brain) ECF [206–208]. Notably, also a distinction between drug binding to specific binding sites and non-specific binding sites (see “Drug binding”) is made [208].

### Modelling drug metabolism in the brain

Drug metabolism within the brain is commonly represented by a loss term, such as an elimination rate constant (s^−1^) [107, 130, 131, 134, 135, 152, 181, 192] or efflux clearance (L s^−1^) [145, 155]. Like BBB transport and cellular uptake, enzyme-mediated metabolic clearance of a drug can be more explicitly described using Michaelis–Menten kinetics [111, 153]:

11 $$\begin{aligned} \Psi _{\mathrm {met}} = V_{\mathrm {max}} \frac{C}{K_{\mathrm {m}}+C}, \end{aligned}$$

with $\Psi$_met_ ($\text {mmol}\; {\text{L}}^{-1}\;\text {min}^{-1}$) the flux of the enzymatic metabolic reaction, *V*_max_ (mmol L^−1^) min^−1^) the maximum flux of this reaction, *C* (mmol L^−1^) the concentration of the substrate (i.e. drug within the brain ECF or brain ICF), and *K*_m_ (mmol L^−1^) the affinity coefficient of the substrate for the enzyme. Equation (1) could be extended as is done in a recent model on brain cellular metabolism [111]. This model accounts for reactions where phosphorylation, oxidation or reduction occurs [111]. There, the reaction flux $\Psi$_met_ defined in Eq. (10) is multiplied with the factor $\frac{r}{v+r}$. In this factor, *r* is the reaction state (i.e. the percentage of metabolites in the phosphorised or reduced state after phosphorylation or reduction by metabolic enzymes) and *v* is a dimensionless affinity factor of the molecule to the reaction [111].

### Modelling drug exchange between compartments

Compartmental models often include several of the processes discussed in the previous sections. The main aim of compartmental models is to predict the concentration-time profile of a drug in several regions, or ‘compartments’, of the body. Typically, compartmental models are pharmacokinetic models that provide a mathematical representation of the exchange of drug between virtual compartments of the body. A compartment represents a body part or drug state (i.e. bound or unbound) that is well-stirred and behaves as one ‘compartment’. Body parts commonly represented by compartments include the blood, entire tissues (e.g. the brain) and components of a tissue (e.g. the brain ECF, the CSF, the brain cells). Transport to, from and between the compartments is described by mass balance equations, usually ordinary differential equations. In general, two assumptions are made:

1. In each compartment, the concentration of drug is homogeneous.
2. The rate of transport between two compartments is proportional to the concentration differences between these compartments and a rate constant (s^−1^) or volumetric clearance ($\text {L}$ s^−1^).

**Fig. 14:**
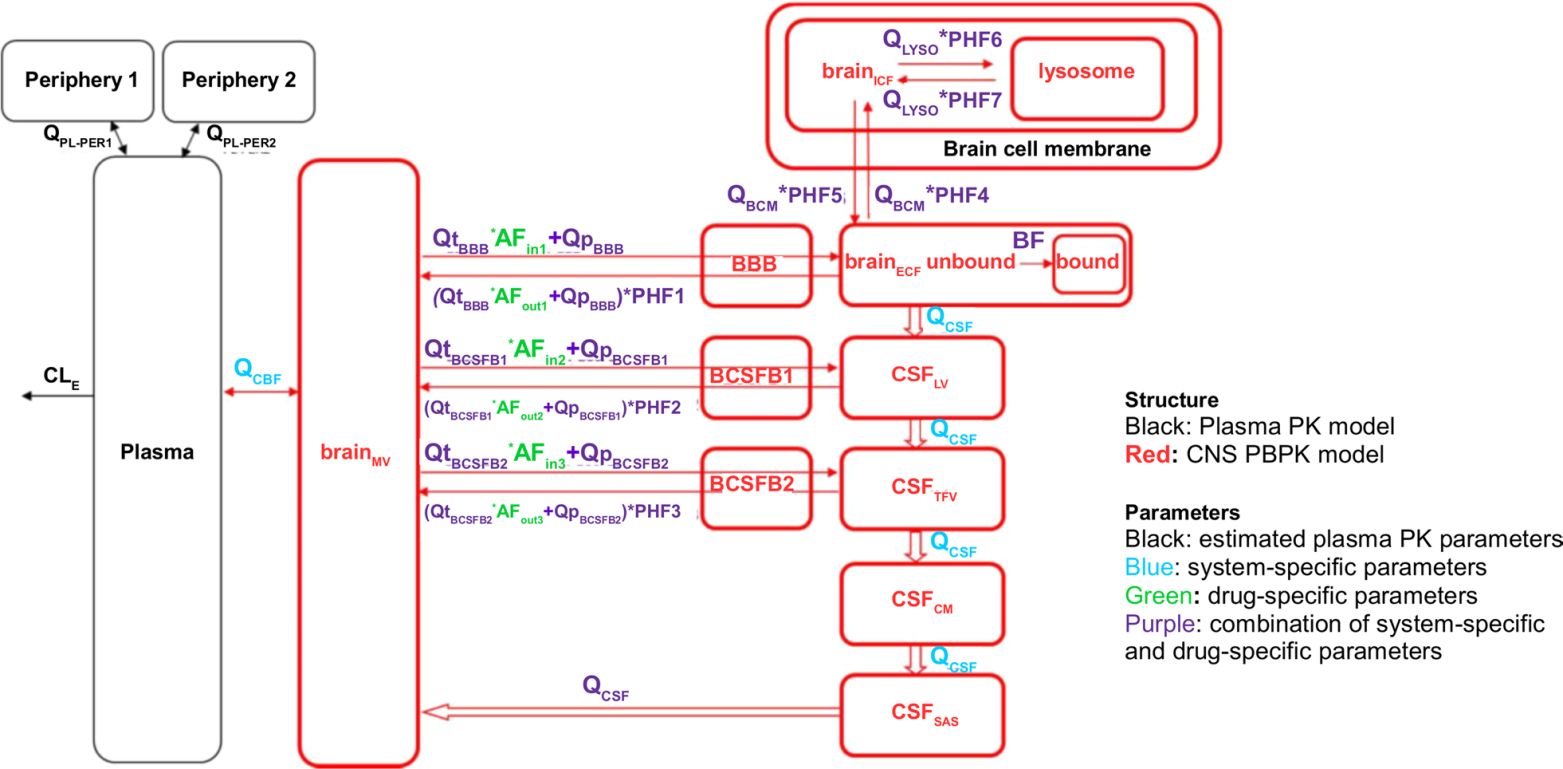
Example of a full physiologically-based pharmacokinetic drug distribution model of the CNS [143]. In this example model, the parameters for the plasma pharmacokinetic model are estimated (black) as input for the full model, while the parameters for the physiologically-based pharmacokinetic model are system-specific (blue) and drug-specific (green). Peripheral compartment 1 and 2 are used in cases where the plasma pharmacokinetic model requires an adequate description of drug concentration in the blood plasma. Here, brain_mv_: brain microvascular, CSF_LV_: CSF in the lateral ventricle, CSF_TFV_: CSF in the third and fourth ventricle, CSF_CM_: CSF in the cisterna magna, CSF_SAS_: CSF in the sub-arachnoid space, Q_CBF_: cerebral blood flow, Qt_BBB_: transcellular diffusion clearance at the BBB, Qp_BBB_: paracellular diffusion clearance at the BBB, Qt_BCSFB1_: transcellular diffusion clearance at the BCSFB, Qp_BCSFB1_: paracellular diffusion clearance at the BCSFB1, Qt_BCSFB2_: transcellular diffusion clearance at the BCSFB2, Qp_BCSFB2_: paracellular diffusion clearance at the BCSFB2, Q_BCM_: passive diffusion clearance at the brain cell membrane, Q_LYSO_: passive diffusion clearance at the membrane of lysosomes, Q_ECF_: brain ECF flow, Q_CSF_: CSF flow, AF_in1-3_: asymmetry factor into the CNS compartments 1-3, AF_out1-3_: asymmetry factor out from the CNS compartments 1-3, PHF1-7: pH-dependent factor 1-7, BF: binding factor. Image by [143] is licensed under CC BY 4.0

Pharmacokinetic models can be empirical or physiologically-based. Empirical pharmacokinetics models are “top down”: a model is developed by improving its fit with available experimental data. This improvement of fit can be established by adding additional compartments or parameters or fine-tuning the values of existing parameters. In contrast, physiologically-based pharmacokinetic models are “bottom-up”: the model is developed based on known physiological data, including both system-specific and drug-specific parameters. The distinction between drug-specific and system-specific parameters allows for a more accurate prediction of drug concentration-time profiles in the different compartments. Moreover, differences between species can be modelled more reliably by setting the parameter values to match the physiological values of the species of interest [209]. Hybrid models exist that integrate empirical pharmacokinetic models and physiologically-based pharmacokinetic models into one model. A detailed sketch of such a model is given in Fig. 14, where the coupling of a empirical pharmacokinetic model of the blood plasma to a physiologically-based pharmacokinetics model of the CNS is shown. There, the parameters for the empirical pharmacokinetic model of the blood plasma are estimated, while the parameters for the physiologically-based pharmacokinetic model of the CNS are experimentally measured brain-specific and drug-specific parameters. Different research questions and different drugs require different compartmental models and parameters. In Table 2, we summarise the brain components that are described by compartments in several studies. As a wide range of compartmental models exists, we thereby limit ourselves to the descriptions of examples we mention in this review. Note that the brain ECF and the brain ICF are sometimes taken together as the brain tissue. Depending on the purpose of the study, other components may be added to the model or one component may be described by more than one compartment. For example, the CSF is often described by multiple compartments, since it is widely distributed over the CNS. In [143], the CSF is described by four compartments where each compartment represents another location of the CSF, see Fig. 14. This study also includes lysosomes as an additional compartment to cover the accumulation of basic drugs in lysosomes (see “Drug extra/intracellular exchange”). In other examples, the blood is described by multiple compartments including those describing the arteries, arterioles, brain capillaries, venules, veins and the sinus sagittalis (a venous channel located between the layers of the dura mater that drains blood from the brain back into the blood) [210, 211]. To develop a compartmental model that best fits its purpose, usually, more models are compared and the model that best fits the experimental data, is chosen [137]. Compartmental models may cover multiple processes affecting the drug distribution within the brain by extending the model. The disadvantage of simple compartmental models, however, is that they do not describe drug distribution *within* the compartments. For that, hybrid models were developed to integrate compartmental exchange with distribution within the brain ECF, as will be discussed below.

**Table 2:** Examples in which combinations of compartments are used

Model	Blood	Brain ECF	Brain ICF	Brain tissue	CSF	Periphery
Collins [212]	1	–	–	1	1	–
Stevens [136]	1	–	–	1	–	1
Jung [210]	4	–	–	1	1	–
Linninger [211]	14	–	–	1	5	–
Gaohua [145]	1	–	–	1	2	10+
Westerhout [137]	1	0.5^a^	0.5^a^	1	4	1
Nhan [126]	1	1	1	–	^b^	–
Ehlers [106]	1	1	–	^c^	–	–
Westerhout [138]	1	1	–	–	2	2
Westerhout [139]	1	1	–	–	4	2
Kielbasa [140]	1	1	1	–	1	–
Ball [142]	1	1	1	–	1	8
Yamamoto [141]	1	1	1	–	4	2
Yamamoto [143]	2	1	2	–	4	2

A wide range of compartmental models exists and therefore we limit ourselves to descriptions of the examples we have mentioned in the text. Compartments include the blood, the brain ECF, the brain ICF, the brain tissue, the CSF and the periphery. The brain tissue represents the brain ECF and the brain ICF together. The periphery refers to components related to other organs than the brain. Numbers indicate the amount of compartments that are used for each component. For example, in the model of Yamamoto [143] (see Fig. 14), two compartments are used for the blood to describe both the blood in the microvasculature (the brain capillaries) and in the larger vessels, while four compartments are used for the CSF to describe the several regions where the CSF resides. Stripes (–) indicate that the component is not described

^a^The brain ECF and brain ICF are modelled as one compartment (the brain tissue)

^b^The CSF clearance is included as a loss term in the description of the brain ECF compartment.

^c^The brain ECF is taken together with the brain extravascular space in one compartment

### Integration of model properties

**Fig. 15:**
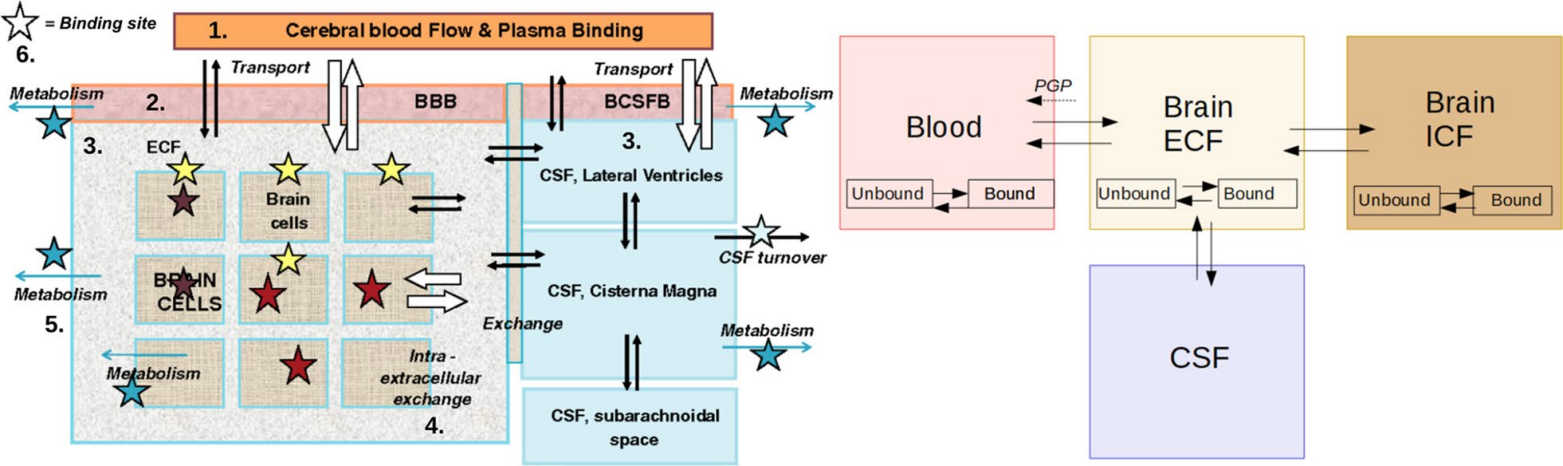
Mathematical representations of drug distribution within the brain. Left: “schematic presentation of the major compartments of the mammalian brain and routes for drug exchange” [45]. The image shows the processes of drug distribution within the brain covered by mathematical models. Processes include cerebral blood flow and plasma binding (see “Modelling drug transport through the brain capillary system”), BBB transport (see “Modelling drug transport across the BBB”), transport within the brain ECF (see “Modelling drug transport within the brain ECF)” intra-extracellular exchange (see “Modelling intra-extracellular exchange”), binding (see “Modelling drug binding kinetics”) and metabolism (see “Modelling drug metabolism in the brain”). The image by [45] is licensed under CC BY 2.0 and modified for the purpose of this review. Right: simplified representation of drug distribution within the brain by a compartmental model. See Table 2 for references. Drug exchange between compartments but not *within* compartments is described (see “Modelling drug exchange between compartments”)

The drug distribution within the brain ECF is affected by many processes. In this section, we first described currently existing mathematical models of the different processes affecting drug distribution within the brain. A sketch of the processes of drug distribution discussed in this review is given in Fig. 15 (left). In the previous subsection (“Modelling drug exchange between compartments”) we discussed compartmental models, that provide a simplified representation of drug distribution within the brain in which exchange between compartments but not *within* compartments is described (Fig. 15, right). Models on drug distribution within the brain ECF (see “Modelling drug transport within the brain ECF”) often include descriptions of other processes affecting drug distribution as have been discussed in this section. In addition, simple compartmental models (that do not include spatial distribution within the compartments, such as the brain ECF) may also cover multiple of the processes affecting drug distribution. Below, we will briefly describe how multiple processes are covered in both classes of models. A summary of this is provided in Table 3. Models on drug distribution within the brain ECF usually describe the BBB with a simple elimination rate constant (see “Modelling drug transport across the BBB”). Only few models take (passive) bidirectional drug transport across a concentration gradient into account [133, 134, 154]. Cellular exchange and metabolism are described with a simple rate constant or using a more extensive description involving saturation of active transporters or metabolic enzymes (see “Modelling intra-extracellular exchange” and “Modelling drug metabolism in the brain”). None of the models on drug transport within the brain ECF includes complete descriptions of drug binding kinetics (see “Modelling drug binding kinetics”). Compartmental models often include passive BBB transport and cellular exchange by describing them as the concentration-dependent, bi-directional transport of drug between the blood and the brain ECF or between the brain ECF and brain ICF compartment, respectively. Some models also include active transport across the BBB [1, 138, 139, 147]. Binding can be described as a distribution of drug between a bound and an unbound compartment (see e.g. [88] and “Modelling drug exchange between compartments”). Metabolism can be quantified by including a rate constant in the model.

#### Hybrid models

Compartmental models are able to cover most of the properties discussed in the previous sections. However, simple compartmental models lack descriptions of the spatial distribution of a drug *within* the compartments. Models that include drug transport *within* the brain ECF and other components of the brain give a more detailed representation of drug distribution within the brain. These models combine models on drug distribution within the brain ECF with compartmental models. Examples of these hybrid models will be described below. Their properties are summarised in Table 3. The model of Collins and Dedrick [212] is one of the earliest models that not only describes drug exchange between the CSF and the brain ECF, but also the drug transport *within* both brain fluids by diffusion and bulk flow [212]. Moreover, the spatial distribution of the brain capillaries is taken into account, from which the drug enters the brain ECF. Ehlers and Wagner [106] have developed a multi-scale model of the human brain tissue. On the microscale, the brain tissue is compartmentalised into the blood, the solid skeleton (consisting of the brain tissue cells and the vascular walls) and the brain ECF. Also, the authors model the brain ECF fluid transport within the brain ECF by bulk flow and diffusion. On the macroscale, all individual properties are homogenized for a reduced and simper representation of drug distribution within the brain ECF. A recent model on brain metabolism (discussed in “Modelling drug metabolism in the brain”) demonstrates the importance of spatial distribution [111]. The model shows that the distance between the synapse and the brain capillaries significantly affects the time course of metabolic fluxes and concentrations of metabolites. Importantly, it also shows the effect of dimension on the model outcome. While a three-dimensional representation is most realistic, it is computationally intensive and expensive. In contrast, a one-dimensional representation is computationally cheap, but less realistic. A reduced two-dimensional model provides a traid-off between computational cost and realism. A recent model on the transport of chemotherapeutics within a brain tumour and its surroundings after convection-enhanced delivery offers a close to complete description of drug distribution within the brain [132]. It describes drug transport over several regions of the brain as well as drug exchange between multiple compartments representing components of the brain. The model takes into account all key processes of drug distribution, such as diffusion, the brain ECF bulk flow, drug efflux to the brain capillaries, and metabolism. However, the binding kinetics are not described and neither is a distinction made between specific and non-specific binding. In conclusion, the hybrid models all include many important factors affecting drug distribution within the brain. However, none of the studies describes all factors and, more importantly, none of them explicitly describes drug binding kinetics.

**Table 3:** Characteristics of models on drug distribution within the brain

Process	Blood flow	BBB transport	Transport within the brain ECF		Cellular exchange	Binding kinetics		Metabolism
Sections:	“Modelling drug transport through the brain capillary system”	“Modelling drug transport across the BBB”	“Modelling drug transport within the brain ECF”		“Modelling intra-extracellular exchange”	“Modelling drug binding kinetics”		“Modelling drug metabolism in the brain”
Model			Diffusion	Bulk flow		Specific	Non-specific	
Brain vasculatue								
[125]	+	*	−	−	−	−	−	*
[116]	+	+	+	−	−	−	−	−
[121]	+	+	+	−	−	−	−	*
Brain ECF								
[133]	+	+	+	−	−	−	−	−
[154]	+	+	+	−	+	−	−	+
[129]	−	*	+	+	+	*	*	*
[71, 150, 150, 156, 182, 182, 184]	−	−	+	−	−	−	−	−
[33, 164]	−	−	+	+	−	−	−	−
[192]	−	−	+	+	−	−	−	*
[127]	−	−	+	+	+	*	*	−
[107]	−	*	−	+	+	*	−	*
[128]	−	*	+	−	−	−	−	−
[135, 152]	−	*	+	−	*	−	−	*
[153]	−	*	+	−	+	*	*	*
[130, 131]	−	*	+	+	−	*	*	*
[134]	−	+	+	−	−	−	*	*
Compartmental								
[142]	+	+	−	−	+	−	*	−
[145]	+	+	−	−	−	−	*	*
[136, 137, 139]	−	+	−	−	−	−	−	−
[141]	−	+	−	−	+	−	−	−
[140, 143]	−	+	−	−	+	−	*	−
Hybrid								
[111]	+	+	+	+	+	−	−	+
[212]	−	−	−	−	−	−	−	−
[106]	−	−	+	+	−	−	−	−
[132]	−	*	+	+	*	*	*	*
[54]	−	+	−	+	+	−	*	−
[126]	−	+	+	+	+	−	*	−

Models are categorised based on their inclusion of processes affecting drug distribution within the brain as discussed in this section. In simple compartmental models, discussed in “Modelling drug exchange between compartments”, transport within compartments (such as the brain ECF) is not described, but multiple of the other processes may be covered. Models on drug distribution within the brain are classified into models on transport to the brain from the brain vasculature, transport within the brain ECF (i.e. models that include spatial transport within the brain ECF), simple compartmental models (i.e. models that include drug exchange between several compartments representing components of or related to the brain tissue but do not include spatial distribution within the compartments) and hybrid models (i.e. models that are a combination of multiple classes)

^+^The process is covered

^−^The process is not covered

* The process is covered, but by an elimination rate constant rather than by a complete description of the process

## The need for a refined mathematical model on spatial drug distribution within the brain

In this review, we have described several classes of models on drug distribution within the brain:

1. Models on processes affecting drug distribution within the brain ECF (described in “Modelling drug transport through the brain capillary system”, “Modelling drug transport across the BBB”, “Modelling drug transport within the brain ECF”, “Modelling intra-extracellular exchange”, “Modelling drug binding kinetics” and “Modelling drug metabolism in the brain”).
2. Models on drug exchange between compartments (described in “Modelling drug exchange between compartments”).
3. Models that integrate multiple properties and/or combine class (1) and (2) (described in "Integration of model properties").

We have touched upon many mathematical models for each class, see Table 3 for an overview. However, none of these models captures all of these processes, while this is crucial for a complete understanding of drug distribution within the brain. Models on drug distribution within the brain often incompletely describe BBB transport and drug binding by simple sink terms that do not take into account bidirectional or saturable transport and binding kinetics. Clearly, a model is needed that covers both drug transport across the BBB and the subsequent distribution within the brain, including drug transport and drug binding. It is important to study the fate of a drug after it crosses the BBB, as many processes within the brain can affect the drug concentration within the brain and thereby influence its effect. For example, a drug can easily pass the BBB, but if subsequent drug transport is slow or if non-specific binding is high, drug-target binding and effect will still be minimal. In “Factors affecting drug distribution within the brain”, we have shown that many factors influence the local distribution of a drug within the brain. Therefore, it is necessary to study the local drug distribution within the brain as well as how this is affected by drug-specific and system-specific parameters. Especially parameters related to drug binding are crucial, as a drug will only exert its desired effect when it is bound to a local target. There is a high demand for approaches that integrate all the aspects discussed in this review into one model. Therefore, a spatial drug distribution model is necessary that predicts the concentration of drug over both time and space and that allows for integration of physiological parameters. In particular, a three-dimensional model is desired, as this will provide the most realistic representation of the three-dimensional brain. The results of such a model can be compared with experimental data of specific drugs in order to gain more insights into the unknowns, including unknown parameter values but in particular the spatial distribution of a drug. Moreover, a model like this can be used to study the influence of regional differences on drug distribution. In summary, setting up a three-dimensional model that integrates BBB transport, drug transport and drug binding within the brain will lead to a more accurate and fine-tuned prediction of drug distribution in the brain.

## Appendix: Ranges of values and units of parameters on drug distribution into and within rat and human brain

Value ranges and units of parameters related to drug distribution into and within the brain are summarized for rat and human in Tables 4, 5, 6, 7, 8 and 9. The tables are in line with the structure of the section “Existing models on the local distribution of drugs in the brain."

**Table 4:** Parameters and units of the brain capillary system. The physiological range of values of the parameters is given for rat and human, based on references from the literature

Parameter	Value (rat)	Value (human)
Capillary radius (10^−6^ m)	0.8–4.8 [4, 5, 7, 213]	3−5 [17, 214, 215]
*S*, capillary surface area (m^2^)	1.3 [216]	15–25 [17, 113, 217]
Intercapillary distance (10^−6^ m)	14–72 [4, 5, 213]	40 [17, 129, 218]–60 [214]
*Q*, capillary blood flow rate (10^−9^ L min^−1^)	4.82–8.83 [219]	0.3–200 [114]
*V*_brain_, brain volume (g)	1.8 [171, 220]	1400 [41]
Capillary blood flow velocity (10^−4^ m s^−1^)	0.5−50 [221–226]	–

**Table 5:** Parameters and units of drug transport across the brain barriers. The physiological range of values of the parameters is given for rat and human, based on references from the literature

Parameter	Value (rat)	Value (human)
*P*, BBB passive permeability (m s^−1^)	10^–10^–10^−5^ [17]	6 × 10^−8^–10^−6^ [227]
*k*_BBB_, BBB rate constant (s^−1^)	3.5 × 10^−5^–1.17 × 10^−3^ [128, 134, 135]	1.4 × 10^−4^–1.4 × 10^−2^ [132]
*CL*_BBB_, BBB transfer clearance (10^−5^ L s^−1^)	0.15–8.5 [143]	113–850 [227]
*P*_trans_, BBB transcellular permeability (10^−7^ m s^−1^)	10^−3^–10^2^ [17]	0.6–10 [227]
*D*_para_, BBB paracellular diffusivity (10^−12^ m^2^ s^−1^)	467–767 [143]	550–767 [227]
*W*_TJ_, width tight junction (10^−6^ m)	0.3–0.5 [143, 228]	0.3–0.5 [227, 228]
*T*_m_, active transporter velocity	10^−5^−10^−8^$\upmu$mol s^−1^ [229]	22–167 $\upmu$mol L^−1^ s^−1^ [111]
*K*_m_, concentration to reach half of *T*_m_ (10^3^ $\upmu$mol L^−1^)	10^−2^−10^1^ [230]	4.5–5 [111]
Surface area BBB (m^2^)	150 × 10^−4^–263 × 10^−4^ [231–233]	12–18 [2, 17]
Surface area BCSFB (m^2^)	25 × 10^−4^–75 × 10^−4^ [232–234]	6–9 [17]

**Table 6:** Parameters and units of drug transport within the brain tissue. The physiological range of values of the parameters is given for rat and human, based on references from the literature

Parameter	Value (rat)	Value (human)
*D**, effective diffusion constant (10^−10^ m^−2^ s^−1^)	0.1–1 [129, 203]	0.1–15 [131, 189, 235]
$\lambda$, tortuosity	1.44–3.5 [69, 168]	1.50–1.77 [69]
$\alpha$, brain ECF volume fraction	0.05–0.47 [168]	0.23–0.49 [69, 111]
*v*_ECF_, brain ECF bulk flow velocity (10^−7^ m s^−1^)	0.5–50 [22, 236]	2–8 [132]
CSF flow rate (10^−7^ L s^−1^)	0.37 [27, 220]	50–67 [237]

**Table 7:** Parameters and units of intra-extracellular exchange. The physiological range of values of the parameters is given for rat and human, based on references from the literature

Parameter	Value (rat)	Value (human)
*k′*, cellular uptake rate (s^−1^)	2.4–19.0 [129]	0.22–304.3 [111]
*CL*_cell_, cellular transfer clearance (10^−6^ L s^−1^)	8 × 10^−2^–2.3 × 10^3^ [143]	4.2 × 10^3^–3 × 10^5^ [227]
*V*_ICF_, volume ICF (L)	1.44 [171]	960 [171]

**Table 8:** Parameters and units of drug binding kinetics. The physiological range of values of the parameters is given for rat and human, based on references from the literature

Parameter	Value (rat)	Value (human)
Association rate constant (μmol L^−1^ s^−1^)	2.8 × 10^−4^–2.8 × 10^2^ [238]	2.8 × 10^−5^–2.8 × 10^2^ [88, 239, 240]
Dissociation rate constant (s^−1^)	2.8 × 10^−7^– 2.8 × 10^−1^[238]	2.8 × 10^−7^–2.8 × 10^0^[88, 239, 240]
Binding site concentration (μmol L^−1^)	1 × 10^−4^– 1 × 10^−2^ [238]	1 × 10^−3^–5 × 10^−1^ [88]

**Table 9:** Parameters and units of drug metabolism in the brain. The physiological range of values of the parameters is given for rat and human, based on references from the literature

Parameter	Value (rat)	Value (human)
Elimination rate constant (10^3^ s^−1^)	4–19 [129]	1.1 × 10^−7^–6.8 × 10^4^ [189, 235]
*V*_max_, maximum reaction flux	2.81 × 10^−5^ μmol min^−1^ g^−1^ [241]	5.76 × 10^2^–1 × 10^9^$\upmu$mol L^−1^ min^−1^ [111]
*K*_m_, affinity constant (10^3^ μmol L^−1^)	0.319 [241]	0.003–528 [111]

## Abbreviations

BBB: blood–brain barrier
BCSFB: blood–cerebrospinal fluid barrier
CNS: central nervous system
CSF: cerebrospinal fluid
ECF: extracellular fluid
ICF: intracellular fluid
